# Silencing miR-202-3p increases MMP-1 and promotes a brain invasive phenotype in metastatic breast cancer cells

**DOI:** 10.1371/journal.pone.0239292

**Published:** 2020-10-01

**Authors:** Rania Harati, Shirin Hafezi, Aloïse Mabondzo, Abdelaziz Tlili

**Affiliations:** 1 Department of Pharmacy Practice and Pharmacotherapeutics, College of Pharmacy, University of Sharjah, Sharjah, United Arab Emirates; 2 Sharjah Institute for Medical Research, University of Sharjah, Sharjah, United Arab Emirates; 3 Department of Medicines and Healthcare Technologies, CEA, Paris-Saclay University, Gif-sur-Yvette, France; 4 Department of Applied Biology, College of Sciences, University of Sharjah, Sharjah, United Arab Emirates; Hungarian Academy of Sciences, HUNGARY

## Abstract

**Background:**

Brain metastasis (BM) is a major cause of morbidity and mortality in breast cancer (BC) and its molecular mechanism remains poorly understood. Transmigration of metastatic cells through the brain endothelium is an essential step in BM. Metalloproteinase-1 (MMP-1) overexpression plays a key role in promoting trans-endothelial migration by degrading the inter-endothelial junctions and disrupting the endothelial integrity. However, little is known about the molecular mechanisms that induce MMP-1 in metastatic cells granting them a brain invasive phenotype. MiR-202-3p is downregulated in brain metastases compared to primary breast tumors and directly targets MMP-1. Here, we unraveled a critical role of miR-202-3p loss in MMP-1 upregulation promoting transmigration of metastatic cells through the brain endothelium.

**Methods:**

A variant of the MDA-MB-231 human BC cell line (MDA-MB-231-BrM2) selected for its propensity to form brain metastases was found to express high levels of MMP-1 and low levels of miR-202-3p compared to the parental cells. Using a gain-and-loss of function approach, we modulated levels of miR-202-3p and examined the resultant effect on MMP-1 expression. Effect of miR-202-3p modulation on integrity of the brain endothelium and the transmigrative ability of BC cells were also examined.

**Results:**

Loss of miR-202-3p in breast cancer cells enhanced their transmigration through the brain endothelium by upregulating MMP-1 and disrupting the inter-endothelial junctions (claudin-5, ZO-1 and ß-catenin). Restoring miR-202-3p exerted a metastasis-suppressive effect and preserved the endothelial barrier integrity.

**Conclusions:**

Our study identified a critical regulatory role of miR-202-3p in brain metastasis and shed light on miR-202-3p/MMP-1 axis as a novel prognostic and therapeutic target that can be exploited to predict and prevent brain metastasis in breast cancer patients.

## Introduction

Brain Metastasis (BM) occurs in 10–30% of patients with metastatic Breast Cancer (BC) and is a major cause of morbidity and mortality in BC [1]. Current treatments are palliative and achieve only a dismal clinical benefit [2]. Hence, the need to develop novel therapeutic strategies to treat or prevent BM is an urgent unmet medical need. However, a major obstacle that hampers the development of effective therapeutic strategies is the lack of knowledge on the molecular mechanisms driving brain metastasis. Understanding the biology of BM and the molecular makeup of breast tumor cells prone to disseminate to the brain will open avenues for both the prediction of patients at high risk to develop BM and the discovery of new drug targets [3].

Brain metastasis is a complex multi-step process that includes epithelial-mesenchymal transition, tumor cells intravasation into the bloodstream or lymphatics, survival in the circulation, extravasation across the Blood-Brain Barrier (BBB), mesenchymal-epithelial transition and finally brain colonization [4–8]. A critical step in BM is the adhesion and transmigration of circulating metastatic cells through the blood-brain barrier (BBB), a process known as extravasation [9–12]. The BBB is part of the neurovascular unit, located at the level of brain capillaries, at the interface between peripheral circulation and the cerebral tissue. It maintains the cerebral homeostasis and forms a formidable obstacle against the entry of xenobiotics and other harmful substances into the brain. The barrier is formed by a monolayer of specialized brain microvascular endothelial cells (BMVEC), tightly attached to each other by tight and adherens junction complexes that greatly restrict the paracellular permeability. These inter-endothelial tight junctional complexes are particularly critical for the barrier integrity and comprise transmembrane proteins (such as occludin, Claudins and Junction-Associated Molecules (JAMs) linked to the actin cytoskeleton through scaffolding proteins (Zonula-Ocludens ZO1, ZO2, ZO3) connected in turn via cingulin dimers to the actin/myosin cytoskeletal system within the cell. The adherens junctional complexes connect adjacent endothelial cells and are composed of transmembrane proteins (cadherins) linked to the actin cytoskeleton by cytoplasmic proteins (such as ß-catenin and plakoglobin) [13]. The BMVEC are also surrounded by a basement membrane, pericytes and astrocytes which altogether contribute to the restricted permeability, making the BBB the most impermeable endothelium in the human body [13–16]. However, how metastatic breast cancer cells extravasate through this formidable barrier to form secondary metastases in the brain remains incomprehensible. A better understanding of the genetic and molecular mechanisms by which cancer cells acquire their transmigrative capacities to cross the BBB will prompt the development of new therapeutic strategies that prevent entry of BC into the brain.

The transmigration of BC cells through the BBB requires a rearrangement and/or reduction of inter-endothelial junctional proteins including occludin, claudin-5 and zonula occludens-1 (ZO-1) [17–20]. The matrix metalloproteinase-1 (MMP-1) was shown to play a critical role in this process. Indeed, MMP-1 is highly expressed in brain metastases compared to primary tumors [21], and its expression is significantly correlated with brain metastasis [22]. In this context, MMP-1 is the only matrix metalloproteinase found to be significantly correlated with breast cancer brain metastasis [23]. MMP-1 was shown to promote trans-endothelial migration of BC cells through the brain endothelium by degrading the inter-endothelial junctions. In addition, knockdown of MMP-1 in brain metastatic cells significantly suppressed brain metastasis *in vivo* whereas ectopic expression of MMP-1 significantly increased the brain metastatic propensity of non-metastatic cells [23, 24]. However, the molecular mechanisms leading to MMP1 overexpression in metastatic BC cells remains to be determined.

A number of recent studies showed that MMP1 expression can be regulated by endogenous micro-RNAs. Micro-RNAs are a class of non-coding RNA that negatively regulate gene expression at the post-transcriptional level leading to inhibition of mRNA translation [25]. MMP1 can be directly targeted and regulated by micro-RNAs including miR-623 in pancreatic cancer cells [26], miR-361-5p in breast cancer [27], miR-330-5p in eosophageal adenocarcinoma [28], miR-222 in fibroblasts and tongue squamous cell carcinoma [29, 30], miR-675 in chondrocytes, miR-526b in skin cells [31] and miR-202-3p in scleroderma fibrosis [32]. Interestingly, analysis with the GEO2R tool [33–35] of the microRNA array cohort data (GSE37407) which contains global miRNA expression profiling of 47 tumor samples from 14 patients with paired samples from primary breast tumors and corresponding distant metastases revealed that three micro-RNAs known to target MMP1 (specifically, miR-202-3p, miR-623 and miR-145) are downregulated in brain metastatic tumors compared to primary tumors [33, 36]. In accordance with these findings, a significant downregulation of miR-202-3p was also reported in a study by Xing et al., where micro-RNA profiling was performed on 7 primary tumors and their paired metastatic lesions in the brain [37]. However, whether loss of miR-202-3p induces MMP-1 expression in BC cells and promote brain metastasis remains to be demonstrated. In this study, we studied the effect of miR-202-3p on the transmigrative capabilities of BC cells. Our results showed that loss of miR-202-3p induce MMP-1 in BC cells and disrupts the brain endothelial integrity to promote tumor cells transmigration through the brain endothelium. Restoring miR-202-3p expression in metastatic BC cells reduces MMP-1 expression and suppress their trans-endothelial migration. These findings suggest that miR-202-3p loss plays a critical role in brain metastasis of breast cancer by upregulating MMP-1 and that the miR-202-3p/MMP-1 axis may represent a potential prognostic and therapeutic target to predict and prevent breast cancer brain metastasis.

## Materials and methods

### Animals

Animal studies were approved by the University of Sharjah (UOS) animal care and use committee and performed in accordance with the UOS directives for animal care. Adult female mice ([C57BL/6J], 8–10 weeks of age) were obtained from Jackson Laboratory (USA), maintained in a pathogen-free and temperature- and humidity-controlled (19−23°C) room at the University of Sharjah under a 12:12 h light-dark cycle and fed a standard diet ad libitum with free access to tap water.

### Cells and cell culture

The human breast carcinoma cell lines MCF-7 and MDA-MB-231 were purchased from American Type Culture Collection (Rockville, MD). MDA-MB-361 breast cancer cell line was purchased from Sigma-Aldrich (Germany). The brain metastatic variant MDA-MB-231-BrM2 and the parental cells MDA-MB-231-TGL were obtained from Dr Joan Massagué (Memorial Sloan-Kettering Cancer Center, New York, USA) [21]. Cells were cultured in Dulbecco’s Modified Eagle Medium (DMEM) supplemented with 10% FBS, 1X L-Glutamine and antibiotics (1000 U Penicillin/1000 U Streptomycin). The immortalized human cerebral microvascular endothelial cells (hCMEC/D3) were purchased from Cedarlane (Tebu-Bio, France) and cultured in EndoGRO™-MV Complete Medium (cat# SCME004, EMD Millipore, USA) supplemented with 1ng/mL FGF-2 (cat# GF003, EMD Millipore) and antibiotics. hCMEC/D3 were grown to confluency on tissue flasks precoated with thin collagen I (cat# 08–115, EMD Millipore) and fibronectin (cat# F1141, Sigma-Aldrich) coating. All cells were maintained in a 95% humidified air and 5% CO2 incubator at 37°C. Cells were regularly examined by microscopy for phenotypic changes and mycoplasma contamination with DAPI staining before their use. Cancer cells with different brain metastatic propensity were characterized by measuring protein expression of three mediators of breast cancer cells transmigration through the brain endothelium (COX-2 (PTGS2, Prostaglandin-Endoperoxide-Synthase-2), ST6GALNAC5 (ST6-*N*-Acetylgalactosaminide-Alpha-2,6-Sialyltransferase-5 and HBEGF (Heparin-Binding Epidermal growth factor-like Growth Factor)) [21].

#### Establishment of *in vitro* brain endothelium monolayer using hCMEC/D3 cell line

hCMEC/D3 cell line was used in the experiments between passage number 30 and 35. 5 × 10^4^ cells were plated on the upper side of a Transwell membrane (growth area 1.12 cm^2^) pre-coated with collagen I and fibronectin. Under these experimental conditions, hCMEC/D3 cells form a confluent and tight monolayer within 72 h with the highest Transendothelial electrical resistance (TEER) values between days 3 and 7 [38, 39].

#### Isolation of primary brain endothelial cells

Brain endothelial cells were isolated as described previously [40, 41]. Five mice brains were extracted, cut sagittally into two halves and the cerebral cortices emptied of white matter. The meninges and the associated vessels were cleaned off by rolling on sterile gauze compress. The tissue was then mechanically homogenized, pelleted by centrifugation at 1500 rpm for 5 min, then digested in HBSS-1% PSN solution containing 1 mg/mL collagenase Dispase, 10 U/μL DNase-I, and 1 μg/mL TLCK (Sigma-Aldrich, Germany) for 1 h at 37°C. Digested tissue was then pelleted by centrifugation at 1500 rpm for 5 min at 4°C and myelin removed by resuspending the pellet in 20% (w/v) BSA in HBSS-1% PSN solution followed by a centrifugation at 2800 rpm for 30 min. The resulting floating white matter and centrifugation medium were then removed carefully, and the microvessel pellets were resuspended and further digested with the enzymatic solution for 1 h at 37°C, then pelleted by centrifugation at 1500 rpm for 5 min. The isolated brain capillaries were then cultured in a 75 cm^2^ flask pre-coated with collagen type IV and fibronectin in the presence of puromycin (2 μg/mL) in the endothelial basal medium (EGM-2MV Endothelial Med BulletKit, cat # CC-3202, Lonza, Switzerland,) containing 0.1% human recombinant epidermal growth factor (hEGF), 0.04% hydrocortisone, 0.1% human recombinant insulin-like growth factor, 0.1% ascorbic acid, 0.1% gentamicin, 0.1% amphotericin-BN, and 5% fetal bovine serum. On the third day, a new endothelial-specific medium without puromycin was added. On day 7, when cells reached about 90% confluency, the purified endothelial cells were frozen for subsequent use.

#### Purity control of collected brain endothelial cells

The purity of collected brain endothelial cells was assessed by measuring the expression of cell-specific marker genes using specific primer for brain endothelial cells (CD31 or PECAM), for glial cells (glial fibrillary acid protein or GFAP), and for pericytes (α-actin). The following mouse primers from Macrogen (South Korea) were used: Pecam1 Mouse qPCR Primer Pair (NM_008816), F: CCAAAGCCAGTAGCATCATGGTC, R: GGATGGTGAAGTTGGCTACAGG; Gfap Mouse qPCR Primer Pair (NM_010277), F: CACCTACAGGAAATTGCTGGAGG, R: CCACGATGTTCCTCTTGAGGTG; Acta2 Mouse qPCR Primer Pair (NM_007392), F: TGCTGACAGAGGCACCACTGAA, R: CAGTTGTACGTCCAGAGGCATAG.

#### Establishment of the in vitro brain endothelium monolayer using primary brain endothelial cells

Primary brain endothelial cells BECs (5 × 10^4^ cells) were plated on the upper side of a collagen and fibronectin-coated polyester Transwell membrane (Costar, pore size 3 μm; growth area 1.12 cm^2^) in endothelial basal medium. Cells were then incubated at 37°C in a 5% CO2 atmosphere. Under these experimental conditions, brain endothelial cells formed a confluent monolayer within 12 days.

### Transendothelial electrical resistance (TEER) measurement

Integrity of the hCMEC/D3 monolayer was monitored by measuring the TEER using an Endohm 12 chamber and an Endohmeter EVOMX (World Precision Instruments). Prior to TEER measurement, the culture media was refreshed and the Transwell inserts were left 20 min at room temperature to exclude interference of temperature. The background electrical resistance from the coated filter and culture media was substracted from each reading. The TEER values were expressed as Ω.cm^2^ (surface area of the Transwell inserts).

### *In silico* bioinformatics analysis

*In silico* bioinformatics analysis was carried out to identify micro-RNAs that directly target the 3'-UTR of MMP1. We searched for the micro-RNAs using four databases: mirTarBase 7.0, miRanda-mirSVR (microRNA.org), and miRDB, TargetScan 7.2 [42].

### Cell transfection

Breast cancer cells were seeded and grown to ~ 70% confluence. For micro-RNA inhibition studies, 30 nM of miR-202-3p inhibitor (Anti-hsa-miR-202-3p miScript miRNA Inhibitor, cat# 219300) or its negative control (miScript Inhibitor Negative Control, cat# 1027271, Qiagen) were transfected using HiPerFect Transfection Agent in culture media for 48h. To introduce mir-202-3p mimic, cells were transfected with either 5nM of miR-202-3p mimic (Syn-hsa-miR-202-3p miScript miRNA Mimic, Cat# 219600, MIMAT0002811: 5 'AGAGGUAUAGGGCAUGGGAA, Qiagen, Germany), or its negative (scrambled) control (AllStars Negative Control, cat# SI03650318, Qiagen), using HiPerFect Transfection Agent (Qiagen, Germany) in culture media for 48h. For MMP1 inhibition, cells were transfected with 30 nM of the ready-to-use MMP1 siRNA (cat # sc-41552, Santa Cruz) using HiPerFect reagent. For co-transfection of MMP1-siRNA/miR-202-3p miRNA inhibitor, cells were co-transfected with 30 nM of MMP1 siRNA and 30 nM of miR-202-3p inhibitor or AllStars negative control using HiPerFect reagent for 48h. Down- and up-regulation of miR-202-3p and MMP1 were assessed by real-time PCR.

### Cell viability assay

Cancer cells were seeded at a concentration of 5 × 10^3^ cell/well in 96-well plate. After 24 h, cells were treated with miR-202-3p mimic (5 nM), inhibitor (30 nM), MMP1 siRNA (30 nM), or their controls as described above. MTT (3-(4,5-dimethylthiazol-2-yl)-2,5-diphenyltetrazolium bromide) assay was performed after 48h; 100 μL MTT (5 mg/ml in culture media; cat # M5655, Sigma Aldrich) was added and the cells were incubated for 4 h. Supernatant was then removed and 50 μL of DMSO (dimethyl sulfoxide) was added to each well. Cell viability was assessed by measuring the absorbance at 540 nm wavelength using MultiskanGo microplate spectrophotometer (Thermofischer, USA).

### Real-time PCR

Total RNAs were isolated from cells using the miRNeasy Micro Kit (cat# 217084, Qiagen, Germany) according to the manufacturer’s protocol. The quantity and quality of the total RNA were controlled using a NanoDrop™ 2000/2000c spectrophotometer (Thermo Scientific, USA). 1 μg of total RNA was used to synthesize single stranded cDNA using the miScript II RT Kit (cat# 218161, Qiagen) for miRNA and the RT2 First Strand Kit (Cat#, 330401, Qiagen) for mRNA. The levels of miR-202-3p and MMP1 were then measured by SYBR Green quantitative real-time PCR. PCR amplifications were performed on the "Applied Biosystems® StepOne™ Real-Time PCR System" using the miScript SYBR Green PCR Kit (Cat# 218075) for miRNA expression and the RT^2^ SYBR Green ROX qPCR Mastermix (cat# 330522) for mRNA expression following the manufacturer’s protocol. The following primers from Qiagen were used: Hs_miR-202_2 miScript Primer Assay (Cat# MS00009037) and RT^2^ qPCR Primer Assay for Human MMP1 (cat# PPH00120B-200). Hs_RNU6-2_11 miScript Primer Assay (cat# MS00033740) and RT^2^ qPCR Primer Assay for Human GAPDH (Cat# PPH72843A) were used as an internal control. The PCR cycling conditions for micro-RNA expression were: 95°C for 15 min followed by 40 cycles of three steps (94°C for 15s, 55°C for 30 s, 70°C for 30s). The PCR cycling conditions for mRNA expression were: 95°C for 10 min followed by 40 cycles of two steps (95°C for 15s, 60°C for 60s). Each assay was performed in triplicate. PCR efficiency was checked prior to the analysis by performing a dilution series experiment using each target assay. The specificity of each reaction was also assessed by melting curve analysis. The relative gene expression was determined using the 2^-ΔCt^ and 2^-ΔΔCt^ methods [43].

### Trans-endothelial migration assay

hCMEC/D3 and primary brain endothelial cells were cultured on the apical side of a collagen and fibronectin-coated transwell insert (3 μm pore size, Corning) until confluency. On the day of the experiment, tightness of the monolayer was monitored by TEER measurements. TEER values of control endothelial monolayers hCMEC/D3 were around 70 Ω.cm^2^ between days 5–7 of culture. Chambers with a TEER lower than 65 Ω.cm^2^ were discarded. TEER values of control primary brain endothelial cells were around 500 Ω.cm^2^ on day 12 of culture. For transmigration assays, transfected or control BC cells were labeled with 5 μM of CellTracker™Green CMFDA fluorescent dye (cat# C2925, Thermofischer-Scientific, USA) for 30 min. 5×10^4^ cells were then added gently on the endothelial monolayer and allowed to transmigrate through the endothelial monolayer for 24h with a PBS washing performed four hours after the co-culture to remove the non-adherent cancer cells. After 24 hours, TEER was measured again and non-migrated cells remaining in the upper chamber were carefully removed with a cotton swab and cancer cells in the basal compartment that have transmigrated through the brain endothelium were washed with PBS and fixed with 4% PFA for 20 min for imaging and counting under Olympus BX43 fluorescent microscope, or lysed with RIPA 1X buffer for fluorescence quantification [44]. The fluorescence signal in the lower compartment was quantified in Varioskan Flash microplate reader (Thermo Fisher scientific) at wavelength excitation/emission: 492/517 nm.

### Cell sorting and western-blot

MDA‐MB‐231-TGL and MDA-MB-231-Brm2 cells are stably transduced with a lentivirus expressing a triple-fusion reporter (TGL) encoding herpes simplex virus thymidine kinase 1, green florescence protein (GFP) and firefly luciferase [21]. Pre-transfected GFP-expressing cancer cells were co‐cultured with hCMEC/D3 cells. After 24h of co-culture, Fluorescence-Activated Cell Sorting (FACS) was performed to separate GFP-expressing cancer cells from unlabeled hCMEC/D3 based on fluorescent labelling. Specifically, cells were collected with accutase (Corning, USA) and sorted with the BD FACSAria™ III cell sorter (BD Biosciences, USA) using the 488 nm laser. hCMEC/D3 cells in mono-culture were also collected and sorted based on the gating parameters used to sort the co-cultured cells. Western-blot analysis was then performed on the collected hCMEC/D3 according to standard procedures to determine protein expression levels of the inter-endothelial junctions (ZO-1, Claudin-5 and ß-catenin). MMP-1 levels in control and transfected cancer cells were also determined by western-blot. Briefly, cells were lysed on ice with RIPA lysis buffer 1X that contains a cocktail of inhibitors (1 mM PMSF, 1 mM sodium orthovanadate, 1 μg/ml leupeptin, and a protease inhibitor cocktail (cat# P2714, Sigma). Subsequently, the lysates were centrifuged at 14000g for 20 min at 4°C. The protein concentrations were determined using the Thermo Scientific Pierce BCA Protein Assay Kit. The cell lysates (20 μg of protein per lane) were diluted in 1X Laemelli’s buffer solution, at 95°C for 5 min. Total protein (20 μg/lane) were separated on 12% SDS-polyacrylamide gels and then transferred onto nitrocellulose membranes (Amersham, Germany) by the wet-transfer system (Bio-Rad, USA). The membranes were blocked with 7.5% skim milk in Tween Tris-Buffered saline (TTBS) for 1h at room temperature and incubated with primary antibodies at 4°C overnight and then with secondary anti-rabbit IgG (1/3000) or anti-mouse IgG (1/3000) antibodies conjugated to horseradish peroxidase for 1 h at room temperature. The blots were then visualized with enhanced chemiluminescence (ECL) kit (using the *ECL™ Prime Western Blotting* System GE Healthcare, cat# RPN2232 or the Bio-Rad Clarity Western ECL Substrate, cat# 1705061) and imaged using ChemiDoc*™* Touch Imaging System (Bio-Rad). The following primary antibodies from Abcam were used: Anti-MMP-1 (ab38929) 1/5000, Anti-Claudin-5 (ab15106) 1/500, Anti-ZO-1 (ab216880) 1/1000, and Anti-ß-catenin (ab32572) 1/3500. Anti-GAPDH (ab9485) 1/7500 was used as loading and transfer control.

### Immunofluorescence

Cells were fixed for 10 min with 4% paraformaldehyde, permeabilized with 0.15% Triton™ X-100 in PBS for 10 min and then incubated with blocking solution, 2% BSA for 1h. MMP-1 expression was detected using anti-human MMP-1 primary antibody (ab38929) 1/500 followed by the secondary antibody donkey F(ab')2 anti-rabbit IgG H&L (Phycoerythrin) (ab7007) 1/200. Samples were them mounted in ProLong® Gold Antifade Reagent with DAPI (cat# 8961, Cell Signaling, USA) and examined with Olympus BX43 fluorescent microscope.

### Enzyme-linked immunosorbant assay (ELISA)

MMP-1 protein levels in cell culture supernatants were quantified by ELISA using the Human Total MMP-1 DuoSet (cat# DY901B, R&D Systems) according to manufacturer’s instructions. The detection limit was 62.5 pg/ml. Signal saturation was observed at concentrations > 4000 pg/ml. The optical density was measured using a Multiskan™ GO Microplate Spectrophotometer (Thermo Scientific, USA) at a wavelength of 450 nm. Averages of the optical densities obtained from the duplicate readings for each standard, control, and sample were calculated. MMP-1 concentrations were then calculated by generating a four parameter logistic (4-PL) curve-fit.

### Dual luciferase reporter assay

For miR target validation, the wild-type (WT) putative binding site of miR-202-3p in the 3′UTR of MMP1 predicted by TargetScan 7.2 (position 127–134 of MMP1 3' UTR), and the mutant (MUT) 3’UTR of MMP1 with the seed region deleted were cloned into pmirGLO Dual-Luciferase miRNA Target Expression Vector (pmirGLO-empty, Promega, USA) downstream of the firefly luciferase gene (XbaI and Nhe sites) to obtain Luc Reporter Constructs. The primers used for cloning 3′-UTR-MMP1 in pmirGLO vector were: MMP1_WT_F 5’CTAGGTCACTGATACACAGAATATAATCTTAT TTATACCTCAGTTTGCATATTTTTTTACTATTTAGAATGT3’; MMP1_WT_R 5’CTAGACATTC TAAATAGTAAAAAAATATGCAAACTGAGGTATAAATAAGATTATATTCTGTGTATCAGTGAC3’ (Wild type). The mutant primers were: MMP1-Mut_F 5’CTAGGTC ACTGATACACA GAATATAATCTTATTTGTTTGCATATTTTTTTACTATTTAGAATGT3’; MMP1_Mut_R 5’CTAGAC ATTCTAAATAGTAAAAAAATATGCAAACAAATAAGATTATATTCTGTGTCAGTGAC3’. MDA-MB-231 and MDA-MB-361 cells were plated at a density of 10^5^ cells/well in a 96-well plate and co-transfected with pmirGLO-MUT (100 ng), pmirGLO-WT (100 ng), miR-101 mimic (20 nM) or negative scrambled control depending on treatments and following Lipofectamine 3000 reagent protocol. Cells were harvested 24 h after transfection and cell lysates were used to sequentially measure the Firefly and Renilla luciferase activities using the Dual-Luciferase® Reporter Assay System analysis (cat# E2940, Promega, USA) according to the manufacturer's instructions. Firefly activities were normalized with Renilla luciferase.

### Statistical analysis

Results are expressed as mean ± SD (standard deviation) of three or four independent experiments with (2–3) replicates each. P values were calculated using independent Student’s t tests or one-way ANOVA followed by Bonferroni post hoc test for multiple comparisons. Differences were considered statistically significant at probability levels of P<0.05(*), P<0.01(**), and P<0.001(***). Calculations and figures were generated using the statistical software GraphPad-Prism 8.2.0.

## Results

### MiR-202-3p expression is attenuated in brain metastatic breast cancer cells and directly targets the 3’UTR of MMP1 mRNA

To identify the micro-RNAs that are specifically involved in the upregulation of MMP-1 in brain metastatic breast cancer cells, we analyzed the clinical micro-RNA array cohort data (GSE37407) which contains global microRNA profiling performed on primary breast tumor samples from 10 patients and their corresponding paired brain metastatic tumors [36]. Analysis with the GEO2R tool of the top 250 microRNAs differentially expressed between the primary and brain metastatic breast tumors revealed that five microRNAs are downregulated in brain metastatic tumors compared to breast primary tumors and also predicted to target MMP1 using four prediction databases: mirTarBase 7.0, miRanda-mirSVR (microRNA.org), and miRDB, TargetScan 7.2 [42]: miR-202-3p, miR-326, miR-623, let-7c, and miR-145 (S1 Table). Three of these micro-RNAs (miR-202-3p, miR-623 and miR-145) are validated to target MMP1 [26, 32, 45]. Interestingly, a significant downregulation of miR-202-3p was also reported by a study by Xing et al., where a micro-RNA profiling was performed on 7 primary tumors and their paired metastatic lesions in the brain [37] (S1 Fig). In this context, it is important to note that data availability on microRNAs expression profile in brain metastatic tumors is scarce due to the difficulty in obtaining tissue specimens from brain lesions.

**Fig 1:**
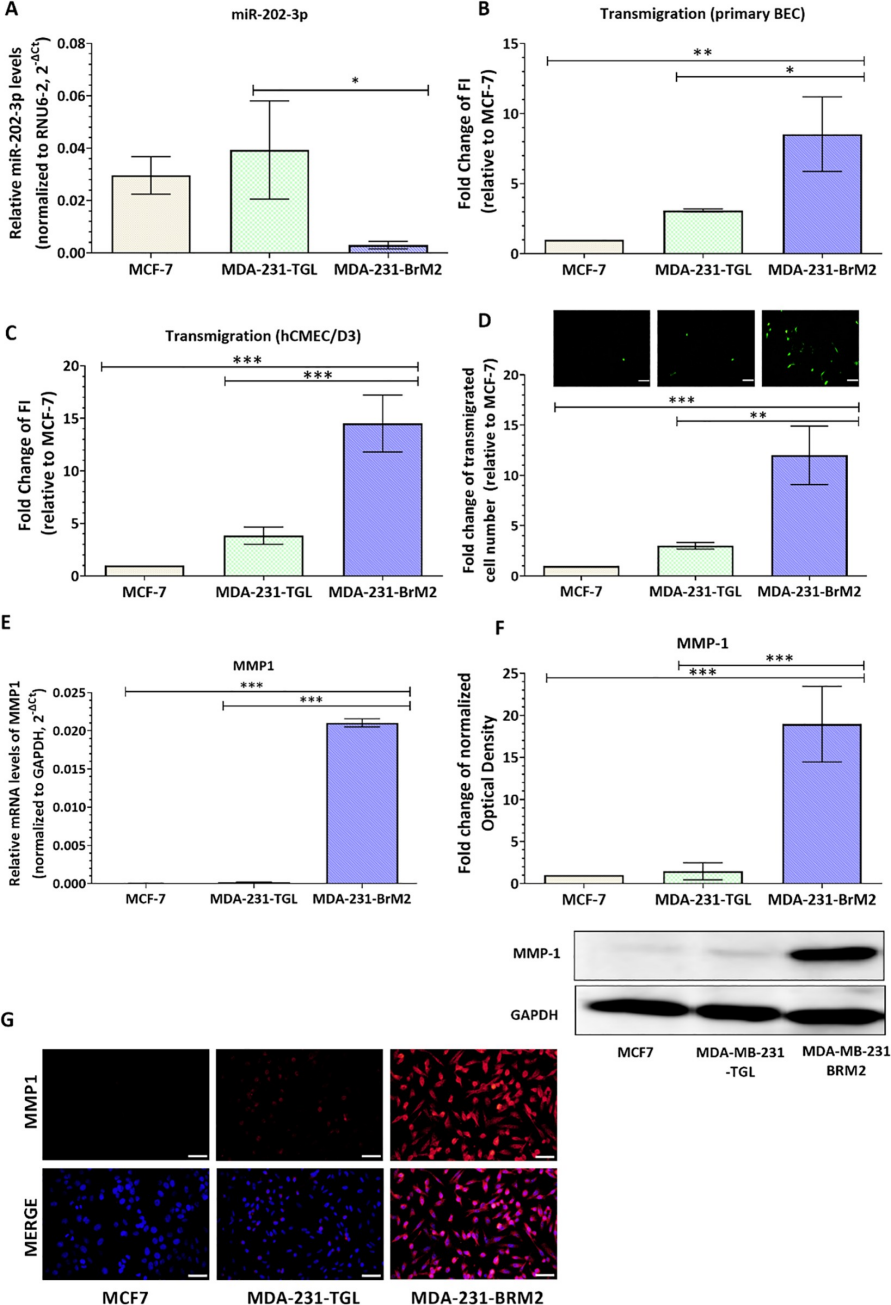
MiR-202-3p is downregulated in brain metastatic breast cancer cell lines and varies inversely with MMP-1 expression. (A) MiR-202-3p relative expression was measured by real-time PCR in three breast cancer cell lines with different brain metastatic propensities (MCF-7; MDA-MB-231-TGL and MDA-MB-231-BrM2). The small nuclear RNA (RNU6-2) was used as an internal standard. Data are represented as 2^^-ΔCt^. (B-D) The transmigrative ability of BC cells through a monolayer of brain endothelial cells was assessed by trans-endothelial migration assay. Cancer cells were labelled with the CellTracker™Green CMFDA fluorescent dye and added on (B) primary brain endothelial cells or (C) hCMEC/D3 monolayer. Four hours later, non-adherent cells were removed by PBS washing. Twenty-four hours following the co-culture, cancer cells that transmigrated to the lower chamber were lyzed with RIPA buffer and fluorescent intensity was measured at excitation/emission: 492/517 nm. Results are expressed as fold change of the fluorescence intensity (FI) relative to MCF-7. The average percentage of plated cells that transmigrated through the layer of primary brain endothelial cells was around 1% for MCF7, 4% for MDA-MB-231-TGL and 15% for MDA-MB-231-BrM2. The average percentage of plated cells that transmigrated through the hCMEC/D3 layer was around 3% for MCF7, 11% for MDA-MB-231-TGL and 42% for MDA-MB-231-BrM2. (D) Cancer cells that transmigrated through the hCMEC/D3 monolayer and the pores of the filter were counted after 24 hours of co-culture in three different fields per insert under fluorescent microscope. Results are expressed as fold change of transmigrated cell number relative to MCF-7. Representative images of transmigrated fluorescently labelled breast cancer cells are shown. The average number of transmigrating cells per field of vision (FOV) ranged from 0–1 for MCF-7, 2–4 for MDA-MB-231-TGL and 8–20 for MDA-MB-231-BrM2. (E) The relative mRNA expression of MMP1 was measured by real-time PCR. GAPDH was used as an internal standard. Data are represented as 2^^-ΔCt^. (F, G) The protein expression of MMP-1 was examined by western-blot (F) and immunofluorescence (G). Optical densities of three independent images were analyzed with Image Lab 6.0.1 software(Bio-Rad) and normalized to GAPDH. Results are represented as fold change of normalized optical densities relative to MCF-7. Experiments were carried out three to four times. Data represent mean ± SD. Scale bar = 50 μm. *p<0.*05*, **p<0.*01*, ***p<0.*001*.

Our preliminary bioinformatics analysis revealed that, among the micro-RNA predicted or validated to target MMP1, miR-202-3p is significantly downregulated in brain metastatic lesions compared to the primary breast tumors [36, 37]. To further validate miR-202-3p downregulation in brain metastatic tumors, we measured miR-202-3p expression in three human BC cells line having different brain metastatic propensities, namely: 1) the non-metastatic MCF-7; 2) the metastatic MDA-MB-231-TGL (MDA231-TGL for brevity); and 3) the brain metastatic MDA-MB-231-BrM2 (MDA231-Br for brevity). MDA231Br is a brain-seeking variant of MDA231 generated by consecutive rounds of *in vivo* selection of clones that metastasized primarily to the brain in 6-7-week old beige nude and athymic mice injected intra-cardially with MDA231 [21]. MCF-7, MDA231-TGL, MDA231-BrM2 were first characterized by assessing the expression of three proteins known to mediate transmigration of BC cells though the brain endothelium (COX-2, ST6GALNAC5 and HBEGF) [21]. Our results confirmed that MDA231-Br express the highest protein levels of these mediators while low levels were detected in MDA231-TGL and no expression was detected in MCF-7 (S2 Fig). MiR-202-3p expression was then measured in BC cells by SYBR green-based real-time PCR. Interestingly, our results showed that miR-202-3p expression was greatly decreased in the MDA231Br cells with higher brain metastatic propensity compared to the less invasive parental MDA231 (p = 0.0260) and MCF-7 cells (Fig 1A). These results are in accordance with the results obtained from patients samples [36, 37]. Fig 1B and 1C shows the transmigration ability of the three BC cell lines through a monolayer of the primary brain endothelial cells (BECs) and hCMEC/D3 cells grown on collagen and fibronectin-coated transwell inserts. Purity of isolated mice brain endothelial cells was verified by measuring expression of markers for brain endothelial cells (CD31 or PECAM), for glial cells (glial fibrillary acid protein or GFAP), and for pericytes (α-actin) as previously described [40, 41]. The purity control showed that the isolated primary BECs consist mainly of endothelial cells as shown by the high levels of PECAM1 (more than 90%) and low levels of GFAP (around 7%) and α-actin (around 1%) (S3 Fig). hCMEC/D3 cells are known to retain the morphological characteristics of primary BECs and form a tight monolayer in culture [38, 39]. Our results show that the MDA-MB-231-BrM2 expressing low levels of miR-202-3p are more capable of crossing the primary brain endothelium and hCMEC/D3 monolayer compared to the parental cells (fold change = 2.76, p = 0.0146 in primary BECs; fold change = 3.8, p = 0.0006 in hCMEC/D3) and the non-metastatic MCF-7 (fold change = 8.5, p = 0.0029 in primary BECs; fold change = 14.5, p = 0.0002 in hCMEC/D3). The number of transmigrated cells was also assessed by counting under fluorescent microscope and our results showed that number of MDA231-BrM2 that transmigrated through hCMEC/D3 layer is higher compared to parental MDA-231TGL (fold change = 4, p = 0.0019) and MCF-7 (fold change = 12, p = 0.0006) (Fig 1D). Interestingly, lowest levels of miR-202-3p were detected in the brain metastatic cells expressing the highest levels of MMP-1. As shown in Fig 1E–1G, MCF-7 and MDA-MB-231 express very low levels of MMP-1, while high MMP-1 levels are detected in the brain metastatic variant. These results indicate that miR-202-2p vary inversely compared to MMP-1 in brain metastatic tumors.

**Fig 2:**
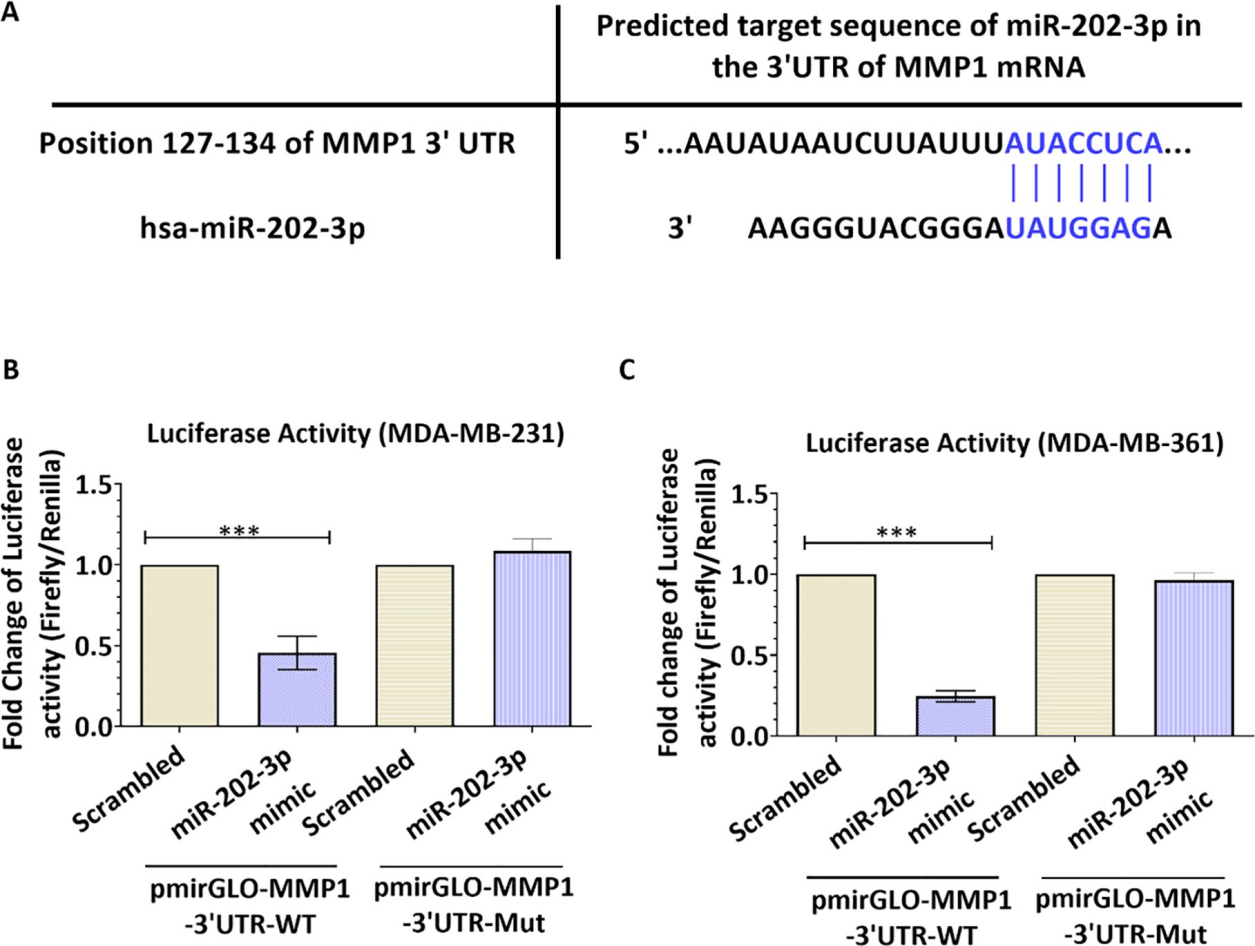
MiR-202-3p directly targets the 3’UTR of MMP1. (A) Schematic representation of the MMP1 3’-UTR with the miR-202-3p binding site. Complementary sequences are represented in blue. (B,C) Effect of miR-202-3p (wild type and mutant) on MMP1 3’UTR luciferase reporters. Constructs carrying MMP1 3’UTR luciferase reporter (pmirGLO-MMP1-3’UTR-WT) or deletion mutant of miR-202-3P binding site (pmirGLO-MMP1-3’UTR-MUT) were co-transfected with miR-202-3p mimic (or negative control) into (B) MDA-MB-231 (ATCC) and (C) MDA-MB-361 cells and subjected to luciferase assays after twenty-four hours. Results are expressed as the fold change of the ratio (firefly to Renilla luciferase activity). Experiments were carried out three to four times. Data represent mean ± SD. *p<0.*05*, **p<0.*01*, ***p<0.*001*.

To determine whether miR-202-3p directly targets the 3’UTR of MMP1 mRNA in metastatic breast cancer cells, we conducted luciferase reporter assays by co-transfection of miR-202-3p mimic and luciferase constructs containing the putative (wild-type WT) or mutated (MUT) binding site of miR-202-3p in the 3′UTR of MMP1. Fig 2A shows the putative target sequence of miR-202-3p in the 3'UTR of MMP1 mRNA as predicted by TargetScan 7.2. The brain metastatic variant MDA-MB-231-BrM2 could not be used for this assay as it is stably transduced with a lentivirus expressing a triple-fusion reporter (TGL) encoding herpes simplex virus thymidine kinase 1, green florescence protein and firefly luciferase. Instead, the MDA-MB-231 (ATCC) and MDA-MB-361 cell line with no endogenous luciferase activities were used for the luciferase assay. Our results showed that Luciferase activity was decreased by approximately 45% in MDA-MB-231 (p = 0.0008, Fig 2B) and by 75% in MDA-MB-361 (p<0.0001, Fig 2C) transfected with miR-202-3p mimic compared to scrambled control in the luc-MMP1 3’UTR WT construct. Moreover, co-transfection of miR-202-3p with a luciferase construct containing the deletion mutant (Luc-MMP1 3’UTR-del) failed to decrease luciferase activity (Fig 2B and 2C). These data are in accordance with previous studies [32] and show that miR-202-3p directly targets MMP1 suggesting a potential role of miR-202-3p in regulating MMP1 in breast cancer brain metastatic tumors.

### Silencing miR-202-3p increases MMP-1 expression in breast cancer cells

**Fig 3:**
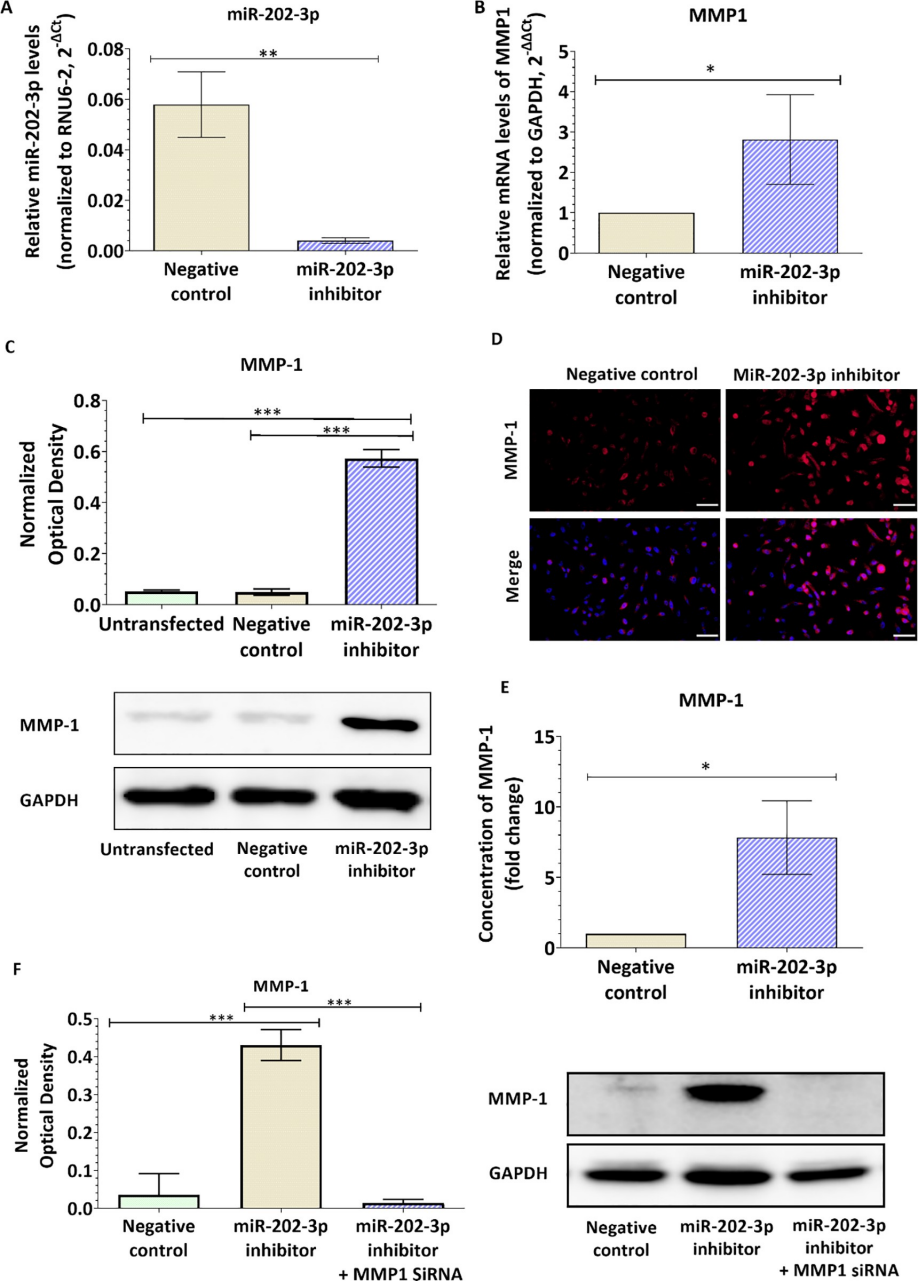
MiR-202-3p suppression enhances MMP-1 expression in breast cancer cells. MDA-MB-231-TGL cells were transiently transfected with miR-202-3p inhibitor or negative control. (A) After 48h, total RNA was extracted and miR-202-3p relative expression was measured by real-time PCR. The small nuclear RNA (RNU6-2) was used as an internal standard. Data are represented as 2^^-ΔCt^. (B) The relative mRNA expression of MMP1 was measured by real-time PCR. GAPDH was used as an internal standard. Data are represented as fold change (2^^-ΔΔCt^) of normalized MMP1 mRNA levels relative to the negative control. (C, D) The protein expression of MMP-1 was examined by western-blot (C) and immunofluorescence (D). Optical densities of three independent images were analyzed with Image Lab 6.0.1 software(Bio-Rad) and normalized to GAPDH. Results are represented as normalized optical densities. (E) MMP-1 levels released in the culture media were quantified by ELISA. Results are represented as fold change of MMP1 concentration levels relative to the negative control. (F) Cells were co-transfected with miR-202-3p and MMP1 siRNA and protein expression of MMP1 was examined by western-blot. Optical densities of three independent images were analyzed with Image Lab 6.0.1 software (Bio-Rad) and normalized to GAPDH. Results are represented as normalized optical densities. Experiments were carried out three to six times. Data represent mean ± SD. Scale bar = 50 μm. *P<0.*05*, **P<0.*01*, ***P<0.*001*.

Our first results showed evidence of reduced levels of miR-202-3p in the brain metastatic breast cancer cells. We also confirmed that miR-202-3p target the 3’UTR of MMP1 mRNA using a dual luciferase reporter gene assay. We next investigated the effect of miR-202-3p loss on the expression of MMP-1 in breast cancer cells. MiR-202-3p levels were ectopically downregulated in the parental MDA-MB-231 cells using a miR-202-3p inhibitor, a chemically modified RNA that specifically inhibit endogenous miRNAs function by complementarity. Transfection of MDA-MB-231 cells with miR-202-3p inhibitor significantly reduced miR-202-3p levels compared to cells transfected with a negative control (p = 0.002, Fig 3A). Effect of miR-202-3p downregulation on MMP1 gene and protein expression was assessed by real-time PCR, and western-blot and immunofluorescence respectively. Results are depicted in Fig 3 and show that loss of miR-202-3p in breast cancer cells significantly increase the gene (fold change = 2.8, p = 0.0476, Fig 3B) and protein expression (fold change = 11.9-fold, p<0.001, Fig 3C) of MMP-1. Downregulation of MMP-1 protein expression was also confirmed by immunofluorescent staining (Fig 3D). In addition, we evaluated the effect of miR-202-3p downregulation on levels of MMP-1 released in the culture media. Total MMP-1 was measured by sandwich ELISA. Our results showed an increase in MMP-1 levels in cells transfected with miR-202-3p inhibitor compared to control cells (fold change = 7.8; p = 0.0105, Fig 3E). Collectively, these data demonstrate that loss of miR-202-3p expression causes a significant increase in MMP-1 expression in breast cancer cells with low brain propensity. To confirm the effect of miR-202-3p on MMP1 and to exclude possible off-target effects, MDA231-TGL cells were co-transfected with miR-202-3p inhibitor and MMP1 siRNA. MMP1 inhibition was confirmed by real-time PCR in MDA231-TGL cells were MMP1 dropped to undetectable levels (data not shown) and in MDA231-BrM2 cells expressing higher levels of MMP1 (S4 Fig). Our results showed that the co-transfection rescued MMP1 knockdown caused by miR-202 inhibitor confirming thus the role of miR-202-3p on MMP1 (Fig 3F). We also evaluated the effect of the different treatments on cell viability by MTT test. Our results showed that the different transfections did not significantly affect cell viability (S5 Fig).

### Loss of MiR-202 in breast cancer cells promotes their transmigration through the brain endothelium and decreases the endothelial barrier integrity

**Fig 4:**
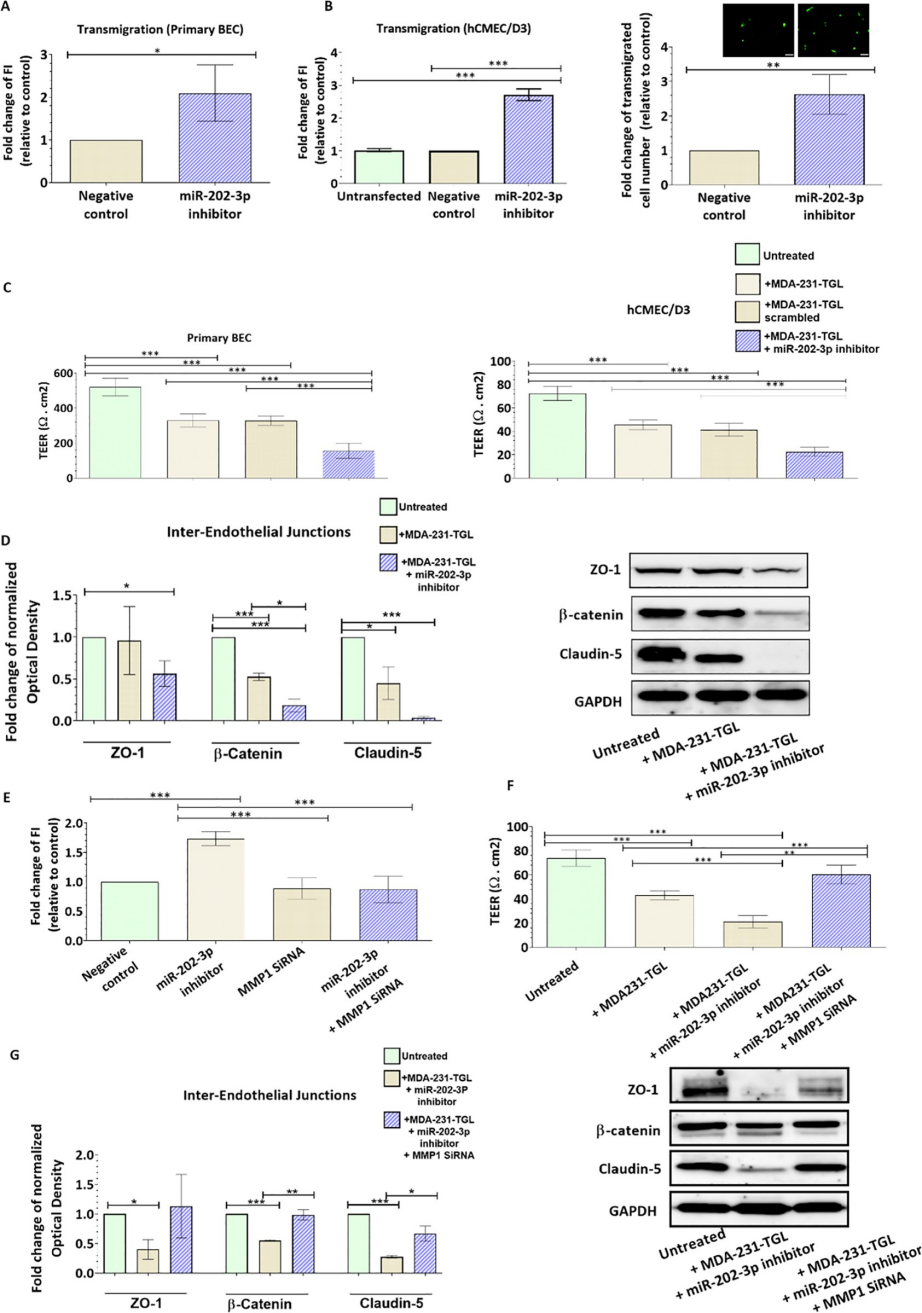
Ectopic downregulation of miR-202-3p in breast cancer cells enhances their transmigration through the brain endothelium and disrupts the endothelial barrier integrity. MDA-MB-231-TGL cells were transfected with miR-202-3p inhibitor (30 nM final concentration) or with negative control. (A,B) The transmigration abilities of the different BC cells were examined by trans-endothelial migration assay. Transfected cancer cells were labelled with the CellTracker™Green CMFDA fluorescent dye and added on (A) primary BECs or on (B) hCMEC/D3 monolayers. Cancer cells that transmigrated to the lower chamber were lyzed with RIPA buffer and fluorescent intensity was measured at excitation/emission: 492/517 nm. Results are expressed as fold change of fluorescent intensity relative to negative control. The average percentage of plated MDA-MB-231-TGL cells that transmigrated through the layer of primary brain endothelial cells was around 4% of control cells and 9% of transfected cells. The average percentage of plated cells that transmigrated through the hCMEC/D3 layer was around 10% of control cells and 27% of transfected cells. (B) Transfected cancer cells that transmigrated through the hCMEC/D3 monolayer and the pores of the filter were counted after 24 hours in three different fields per insert under fluorescent microscope. Results are expressed as fold change of transmigrated cell number relative to MCF-7. Representative images of transmigrated fluorescently labelled MDA-MB-231-TGL (small green cells) in control and anti-miR-202-3p transfected cells are shown. The average number of transmigrating cells per field of vision (FOV) ranged from 1–3 for control cells and 5–10 for transfected cells. (C) Transendothelial electrical resistance (TEER) was measured for brain endothelial cells co-cultured with MDA-MB-231-TGL cells treated with miR-202-3p inhibitor or negative control after 24 hours of co-culture. (D) Protein expression of the inter-endothelial junctions (ZO-1, ß-catenin and claudin-5) was measured by western-blot in hCMEC/D3 co-cultured with MDA-MB-231-TGL cells treated with miR-202-3p inhibitor or control. (E) Fold change of the fluorescent intensity of transmigrated MDA-231-TGL cells pre-transfected with miR-202-3p inhibitor and/or MMP1 SiRNA or negative control. (F) TEER values of hCMEC/D3 cells co-cultured with MDA-MB-231-TGL cells treated with miR-202-3p inhibitor and/or MMP1 SiRNA or negative control after 24 hours of co-culture. (G) Protein expression of the inter-endothelial junctions (ZO-1, ß-catenin and claudin-5) protein expression measured by western-blot in hCMEC/D3 co-cultured with MDA-MB-231-TGL cells treated with miR-202-3p inhibitor and/or MMP1 siRNA or negative control. Optical densities of three independent images were analyzed with Image Lab 6.0.1 software (Bio-Rad) and normalized to GAPDH. Data are mean ±SD from three independent experiments. *p<0.*05*, **p<0.*01*, ***p<0.*001*.

We next investigate whether MMP-1 induction by miR-202 downregulation increases the invasiveness of BC cells through the brain endothelium, key component of the BBB. We examined the trans-endothelial migration ability of MDA231-TGL cells through the monolayer of primary brain endothelial cells and hCMEC/D3 cell line. Our results showed that transfection with miR-202-3p inhibitor increases the transmigration of MDA-MB-231-TGL cells through the brain endothelium compared to control cells (fold change = 2.09, p = 0.0453 in primary BEC; fold change = 2.7, p <0.0001 in hCMCE/D3, Fig 4A and 4B). The number of transmigrated cells was also estimated by counting under fluorescent microscope and results showed an increase in transmigration following miR-202-3p inhibition (fold change = 2.6, p = 0.008, Fig 4B). These results indicate that loss of miR-202-3p in breast cancer cells promotes the trans-endothelial migration of cancer cells.

Transmigration of metastatic cancer cells through the brain endothelium has been associated with degradation of inter-endothelial junctions and permeabilization of the endothelial barrier [17–20]. Therefore, we next investigated the effect of miR-202-3p downregulation and MMP-1 overexpression on integrity of the brain endothelium. TEER of primary BECs and hCMEC/D3 monolayer were measured to assess the barrier integrity. TEER values in monoculture were around 500 Ω.cm^2^ in primary BECs and 70 Ω.cm^2^ in hCMEC/D3 cells. These values are consistent with previous studies where similar ranges were reported as indicators of junctional tightness [38, 39, 46–48]. Co-culture of BECs with MDA231-TGL cells transfected with negative control reduced the TEER by approximately 36 and 42% (p<0.001) in primary BEC and hCMEC/D3 respectively compared to BECs monoculture, while co-culture with MDA231 pretransfected with miR-202-3p inhibitor further reduced the TEER by approximately 33% and 27% (p<0.001) in primary BEC and hCMEC/D3 respectively (Fig 4C). We also examined the protein expression of inter-endothelial junctions (claudin-5, ZO-1 and the adherens junctional protein ß-catenin). Our results show that co-culture of hCMEC/D3 with MDA231-TGL cells transfected with the negative control reduces expression of the inter-endothelial junctions compared to the control hCMEC/D3 (ß-catenin (p<0.001) and Claudin-5 (p = 0.074)), while co-culture of hCMEC/D3 with MDA231 cells transfected with miR-202-3p inhibitor further reduces the protein expression of inter-endothelial junctions (ZO-1 (p = 0.0461), ß-catenin (p<0.001), Claudin-5 (p<0.001)) (Fig 4D). In addition, co-transfection of MDA231-TGL cells with miR-202-3p and MMP1 siRNA rescued the effect of miR-202-3p inhibition on the transmigrative abilities of cancer cells (Fig 4E) and restored normal TEER values and junctions expression similar to the control group in the brain endothelium (Fig 4F and 4G). Taken together, our data demonstrated that loss of miR-202-3p facilitates their transmigration through the brain endothelium by upregulating MMP-1 and degrading the inter-endothelial junctions. These data shed light on the important role played by miR-202-3p loss in the extravasation of breast cancer cells through the brain endothelium, a key step in brain metastasis.

### Restoration of MiR-202-3p expression inhibits MMP-1 expression in brain metastatic breast cancer cells and suppress their trans-endothelial migration by preserving the brain endothelium integrity

**Fig 5:**
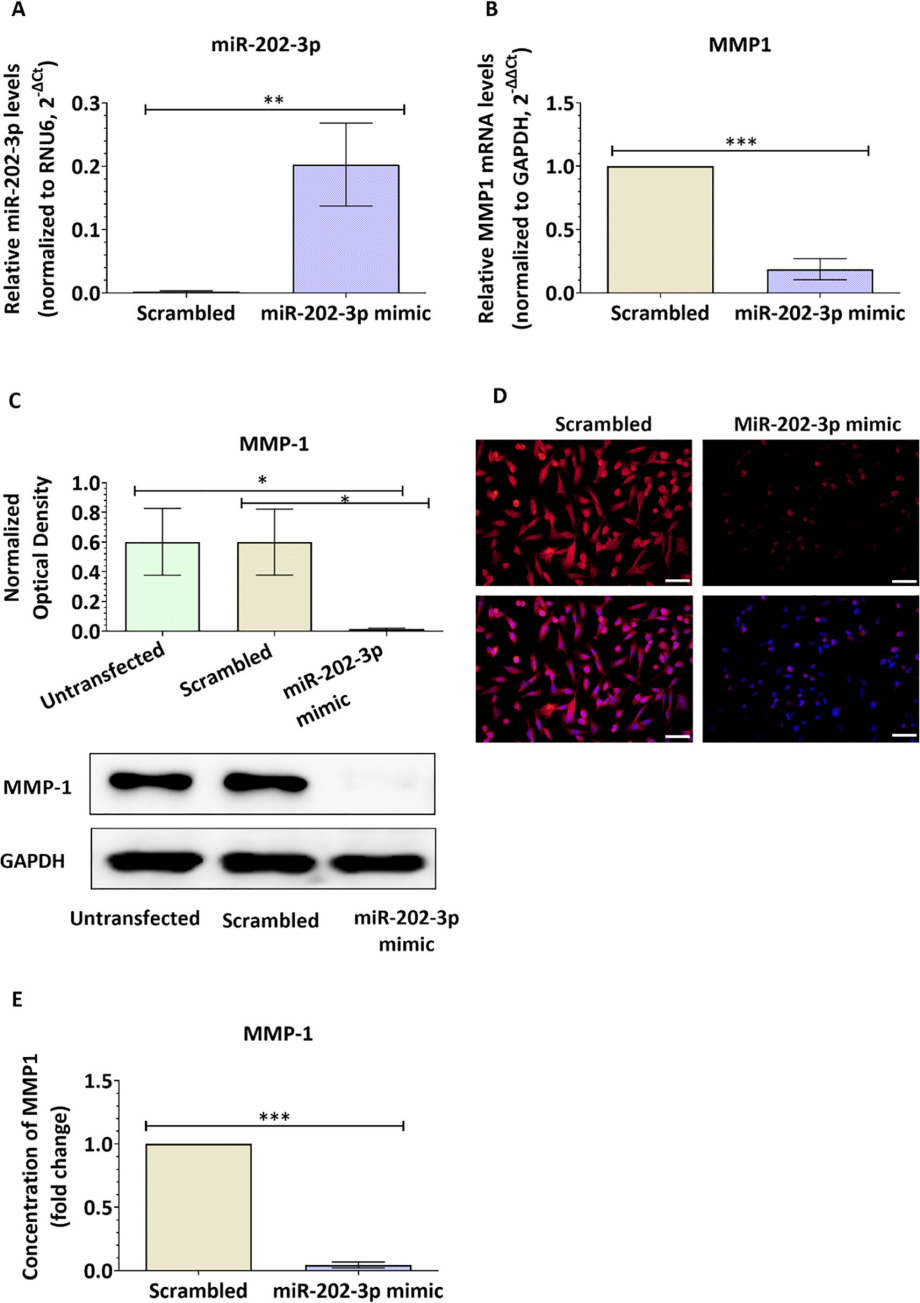
MiR-202-3p upregulation downregulates MMP1 expression in brain metastatic breast cancer cells. MDA-MB-231-BrM2 cells were transiently transfected with miR-202-3p mimic or scrambled control. (A) After 48h, total RNA was extracted and miR-202-3p relative expression was measured by real-time PCR. The small nuclear RNA (RNU6-2) was used as an internal standard. Data are represented as fold change (2^^-ΔCt^) of normalized miR-202-3p levels relative to the control (scrambled). (B) The relative mRNA expression of MMP1 was measured by real-time PCR. GAPDH was used as an internal standard. Data are represented as fold change (2^^-ΔΔCt^) of normalized MMP1 mRNA levels relative to the negative control. (C, D) The protein expression of MMP-1 was examined by western-blot (C) and immunofluorescence (D). Optical densities of three independent images were analyzed with Image Lab 6.0.1 software (Bio-Rad) and normalized to GAPDH. Results are represented as normalized optical densities. (E) MMP-1 levels released in the culture media were quantified by ELISA. Results are represented as fold change of MMP1 concentration levels relative to the control (scrambled). Experiments were carried out three times. Data represent mean ± SD. Scale bar = 50 μm. *p<0.*05*, **p<0.01 and ***p<0.*001*.

**Fig 6:**
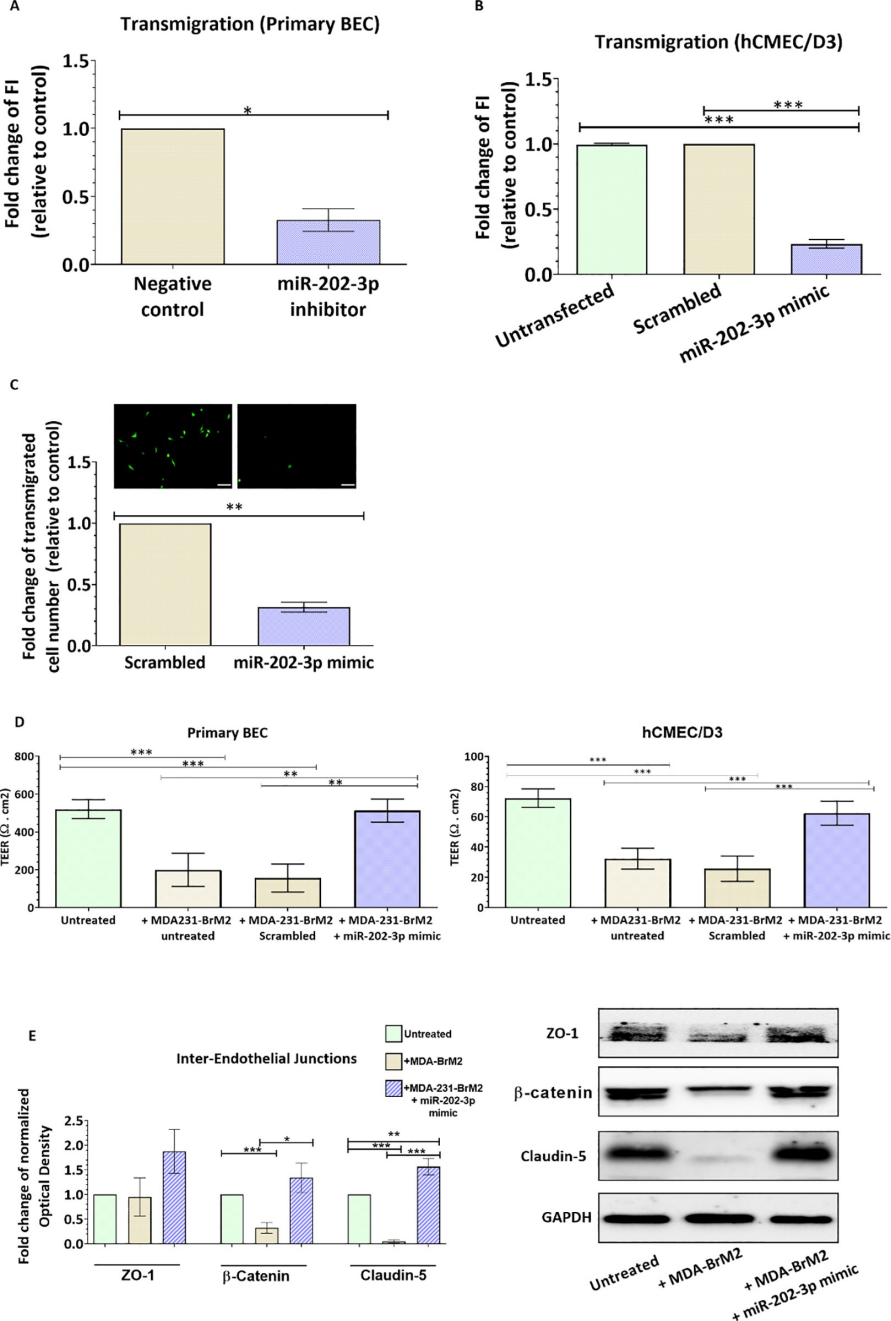
Ectopic upregulation of miR-202-3p in breast cancer cells suppresses their transmigration through the brain endothelium and preserves the endothelial barrier integrity. MDA-MB-231-BrM2 cells were transfected with miR-202-3p mimic or scrambled control. (A-C) The transmigration abilities of the different BC cells were examined by trans-endothelial migration assay. Transfected cancer cells were labelled with the CellTracker™Green CMFDA fluorescent dye and added on (A) primary BECs or (B) hCMEC/D3 monolayer. Results are expressed as fold change of fluorescent intensity relative to the control (scrambled). The average percentage of plated MDA-MB-231-BrM2 cells that transmigrated through the layer of primary brain endothelial cells was around 18% of control cells and 6% of transfected cells. The average percentage of plated cells that transmigrated through the hCMEC/D3 layer was around 39% of control cells and 9% of transfected cells. (C) Transfected cancer cells that transmigrated through the endothelial monolayer and the pores of the filter were counted after 24 hours in three different fields per insert under fluorescent microscope. Results are expressed as fold change of transmigrated cell number relative to MCF-7. Representative images of transmigrated fluorescently labelled MDA-MB-231-BrM2 (small green cells) in control and miR-202-3p transfected cells are shown. The average number of transmigrating cells per field of vision (FOV) ranged from 8–14 for control cells and 2–5 for transfected cells. (D) Trans-endothelial electrical resistance (TEER) was measured for brain endothelial cells co-cultured with MDA-MB-231-BrM2 cells treated with miR-202-3p mimic or scrambled control. (E) Western-blot analysis of the inter-endothelial junctions (ZO-1, ß-catenin and claudin-5) protein expression was measured by western-blot in hCMEC/D3 co-cultured with MDA-MB-231-BrM2 cells treated with miR-202-3p mimic or scrambled control. Optical densities of three independent images were analyzed with Image Lab 6.0.1 software (Bio-Rad) and normalized to GAPDH. Results are represented as fold change of normalized optical densities relative to the control (scrambled). Data are mean ±SD from three to five independent experiments. *p<0.*05*, **p<0.*01*, ***p<0.*001*.

We showed evidence of reduced levels of miR-202-3p in brain metastatic breast cancer cells, and that loss of miR-202-3p induces MMP-1 expression, disrupts the brain endothelium integrity and promotes transmigration of BC cells. We next investigated whether restoring miR-202-3 in metastatic cells could suppress their extravasation through the brain endothelium. MiR-202-3p levels were ectopically induced in the brain metastatic cells MDA231Br by transfection with miR-202-3p mimic, a synthetic double-stranded RNA that stimulates naturally occurring mature miR-202-3p. Transfection with miR-202-3p significantly increased miR-202-3p expression in the metastatic cells when compared to control cells (scrambled miR) (p = 0.006, Fig 5A). To further clarify the mechanism, we examined the effect of miR-202-3p upregulation on MMP-1 expression. Our data showed that transfection of MDA231Br with miR-202-3p mimic greatly reduces MMP1 gene levels (by around 80% (p<0.001, Fig 5B) and inhibited protein levels as shown in Fig 5C and 5D. In addition, miR-202-3p upregulation inhibited levels of MMP-1 released in the culture media as shown by the ELISA results (p<0.001, Fig 5E). MiR-202-3p ectopic induction also reduced the number of cancer cells that had migrated through the primary brain endothelial cells by 68% (p = 0.0001) and through the hCMEC/D3 monolayer by 77% (p<0.0001) compared to control cells (scrambled miR) expressing low levels of miR-202-3p (Fig 6A–6C). The brain endothelium integrity was also examined. Our data showed that TEER values were greatly reduced in primary BECs and hCMEC/D3 cells co-culture with the brain metastatic MDA231Br (65%, p<0.001 and 63%, p<0.001 respectively), while no significant changes in TEER values were observed in brain endothelial cells co-cultured with MDA231Br cells pre-transfected with miR-202-3p mimic (Fig 6D). In accordance with these observations, protein levels of the inter-endothelial junctions (ZO-1, ß-catenin and claudin-5) remained unchanged in hCMEC/D3 co-cultured with MDA231Br cells expressing high levels of miR-202-3p as shown in Fig 6E. We examined whether miR-202-3p inhibitor or mimic could be transferred from cancer cells to the brain endothelium and whether miR-202-3p could directly target the 3’UTR of ZO-1, ß-catenin and claudin-5 mRNAs. hCMEC/D3 cells were co-cultured with control or pre-transfected cancer cells. Twenty-four hours later, cells were sorted by FACS and hCMEC/D3 collected. miR-202-3p expression was examined by real-time PCR. Our results showed that miR-202-3p is not expressed in hCMEC/D3 (Ct around 35) and these levels remained unchanged following the co-culture with transfected cancer cells. In addition, *in silico* bioinformatic analysis (using four databases: mirTarBase 7.0, miRanda-mirSVR (microRNA.org), and miRDB, TargetScan 7.2) did not identify ZO-1, ß-catenin and claudin-5 mRNA as direct targets of miR-202-3p. Taken together, our data show that restoration of miR-202-3p levels directly target and reduce MMP-1 levels in the brain metastatic breast cancer cells which preserves the brain endothelium integrity and prevents extravasation of tumor cells.

## Discussion

In the present study, we provided first-time evidence that loss of miR-202-3p in breast cancer cells induces MMP-1 expression which grants tumor cells a higher brain invasive phenotype allowing them to induce alteration in the brain inter-endothelial junctions and to transmigrate though the brain endothelial barrier. Ectopic restoration of miR-202-3p expression downregulates MMP-1 expression, reduces extravasation of brain metastatic breast cancer cells and preserves the barrier integrity. These finding help in understanding the molecular makeup of metastatic cells more prone to disseminate to the brain and also in the prediction of patients at high risk to develop BM.

Extravasation of breast cancer cells through the brain endothelium is a key step in brain metastasis. During extravasation, metastatic cells release high levels of MMP1 capable of degrading the inter-endothelial junctions to allow paracellular transmigration through the brain endothelium [6, 10, 12, 17, 18, 49]. Due to its important role in tumor metastasis, MMP-1 represents a potential pharmacological target. Several synthetic inhibitors of MMP-1 were developed and trialed in various cancer types (reviewed in [50]). One of the first MMPs inhibitors developed was Batimastat. Batimastat is a multi-MMPs inhibitor that inhibit a broad spectrum of MMPs (including MMP-1, -2, -3, -7, -9, -14, and ADAM-10 and -17). Batismastat showed promising preclinical anti-tumor effect, however significant toxicity was observed [51, 52]. Later, Marimastat, a next-generation analogue with similar mechanism was developed. Marimastat also showed inhibition of multiple MMPs (including MMP-1, -2, -3, -7, -9, and ADAM-10 and -17). Similar to Batimastat, Marimastat also showed promising anti-tumor effect in the preclinical setting. It reached phase II and III clinical trials for its anti-metastatic effect exerted on multiple solid tumors including breast, brain, colorectal, pancreatic and prostate cancer. However, the clinical trials failed to prove a survival benefit; in addition, Marimastat had severe side effects on the musculoskeletal system [53–57]. Later, more selective inhibitors were developed and trialed such as rebimastat, an inhibitor of MMP-1, -2, -3, -8, -9, -13, -14. Rebimastat was tested for the treatment of metastatic lung, breast, and prostate carcinomas. However, the trials failed to demonstrate a positive effect on survival and were cancelled in phase III [58–60]. There are several possible reasons that can explain the failure of MMPIs in clinical trials. Indeed, some MMPs were shown to have anti-tumor effects, and therefore, the MMPIs with a broad spectrum might block these MMPs and result in tumor progression. In addition, MMPs exert important physiological roles, particularly on extracellular matrix remodeling, and therefore inhibiting these physiological roles lead to the unwanted side effects observed with virtually all MMPIs. Furthermore, the clinical trial on MMPIs were performed in patients with advanced metastatic disease, however, MMPs are involved in the early stages of metastasis and MMPIs are expected to be effective in these early stages; therefore, this could potentially explain the lack of efficacy [50, 61]. Taken together, these reasons emphasize the need to develop more tissue- and time-specific MMPIs that should be tested for their anti-metastatic properties in the early pre-metastatic stages where they are expected to exert their therapeutic effect. However, a major obstacle hindering the development of specific MMP inhibitors for clinical use in cancer patients is our lack of knowledge and understanding of the complex MMPs biology and the role played by MMPs in carcinogenesis and metastasis. Furthermore, the molecular mechanisms that induce MMP1 expression to grant tumor cells a higher brain invasive phenotype remain poorly understood. A better understanding of MMPs biology and regulation will prompt the development of more specific and effective MMP inhibitors.

Our findings demonstrated a critical role of miR-202-3p in the regulation of MMP-1 in metastatic BC cells and identified miR-202-p as potential target for better pharmacological interventions. Increasing numbers of studies have shown that micro-RNAs are deregulated in metastatic tumors compared to primary tumors and play key roles in metastasis. For instance, miR-509 was shown to be downregulated in brain metastases and promotes breast cancer cell invasion by upregulating RhoC/MMP9 and TNFα [37]. MiRNA-1258 is also involved in brain metastasis by modulating the expression and activity of heparanase, an enzyme with pro-tumorigenic, pro-angiogenic and pro-metastatic properties [62]. In another study, brain metastatic cancer cells were shown to release microRNA-181c-containing extracellular vesicles capable of destructing the BBB [63]. In addition, we recently demonstrated that loss of miR-101-3p promote transmigration of BC cells by inducing COX-2/MMP1 signaling [64]. These observations point to miRNA as promising therapeutic targets, however, more research is still needed to unravel the panel of specific micro-RNA profiles that regulate the different steps of breast cancer brain metastatic cascade and that are suitable as therapeutic agents to suppress or prevent BM. In this context, none of the micro-RNA deregulated in brain metastatic tumors were tested for their ability to regulate MMP-1, a key player in the transmigration of tumor cells trough the brain endothelium. We found that miR-202-3p is downregulated in brain metastatic cancer cells which increases expression of MMP1, MMP1 being a direct target of miR-202-3p. The high levels of MMP1 released by metastatic cells disrupt the inter-endothelial junctions to promote transmigration of cancer cells through the brain endothelium. These results imply that breast tumor cells expressing low levels of miR-202-3p are at high risk of disseminating to the brain due to the induction of MMP1.

MiR-202 has been associated with several types of cancer and was shown to exert tumor-suppressive effect. For instance, miR-202 was shown to suppress growth and metastasis in prostate cancer by targeting PIK3CA [65]. miR-202 was shown to inhibit cell proliferation and invasion of colorectal cancer and bladder cancer by targeting UHRF1 and EGFR respectively [66, 67]. In breast cancer, MiR-202-3p was shown to act as tumor suppressor [68] and to inhibit proliferation and migration of breast cancer cells by targeting ROCK1 gene [69]. MiR-202-3p was also shown to mediate resistance to doxorubicin through regulation via PI3K/AKT signaling [70]; however, the role of miR-202 have not been investigated in brain metastasis of breast cancer cells. In our study, we unraveled a role of miR-202-3p in brain metastasis. We showed that loss of miR-202-3p in cancer cells with low metastatic propensity increases MMP-1 expression and grants them a brain invasive phenotype rendering these cells more prone to disseminate to the brain. Our data also showed that restoring miR-202-3p expression in brain metastatic cells inhibited MMP-1, protected integrity of the brain endothelial barrier and suppressed the trans-endothelial migration of metastatic cells. These results imply that miR-202-3p is a potential therapeutic target to prevent brain metastasis in breast cancer patients, to date incurable.

## Conclusion

Our study demonstrates the role of miR-202-3p in controlling transmigration of breast cancer cells through the brain endothelium, a key step in BCBM. The results highlight that exogenous modulation of miR-202-3p can attenuate the trans-endothelial migration of metastatic breast cancer cells through reduction of MMP-1 signaling. These results imply that miR-202-3p is a potential prognostic and therapeutic target that can be exploited to predict and prevent spread of breast cancer to the brain in patients at high risk to develop brain metastasis.

## Supporting information

S1 Table Mature micro-RNAs downregulated in GSE37407 and validated or predicted to target MMP1. The clinical micro-RNA array cohort data (GSE37407) which contains global microRNA profiling performed on primary breast tumor samples from 10 patients and their corresponding paired brain metastatic tumors was analyzed with the GEOR tool [33, 36]. Analysis with the GEO2R tool of the top 250 microRNAs differentially expressed between the primary and brain metastatic breast tumors revealed that five microRNAs are downregulated in brain metastatic tumors compared to breast primary tumors and also predicted to target MMP1 using four prediction databases: mirTarBase 7.0, miRanda-mirSVR (microRNA.org), and miRDB, TargetScan 7.2 [42]: miR-202-3p, miR-326, miR-623, let-7c, and miR-145. Three of these micro-RNAs (miR-202-3p, miR-623 and miR-145) are validated to target MMP1 [26, 32, 45]. A significant downregulation of miR-202-3p was also reported by a study by Xing et al., where a micro-RNA profiling was performed on 7 primary tumors and their paired metastatic lesions in the brain [37].
(DOCX)

S1 Fig MiR-202-3p expression in primary and brain metastatic tumors from breast cancer patients (GSE37407) [36, 71, 72]. (PNG)

S2 Fig Expression of C0X-2, ST6GALNAC5 and HBEGF in breast cancer cell lines. COX-2, ST6GALNAC5 and HBEGF protein expression was assessed by western-blot in three breast cancer cell lines with different metastatic propensities (MCF-7; MDA-MB-231-TGL and MDA-MB-231-BrM2).
(TIF)

S3 Fig Expression of CD31, GFAP and α-actin in isolated primary BECs. Gene expression of CD31 (marker of endothelial cells), GFAP (marker of astrocytes) and α-actin (marker of pericytes) was measured by real time PCR in primary BECs isolated from mice.
(TIF)

S4 Fig Knockdown of MMP1 gene expression by MMP1 SiRNA in MDA-MB-231-BrM2. MDA-MB-231-BrM2 cells were treated with MMP1 SiRNA and gene expression was examined by real-time PCR 48 hours later. Experiments were carried out three times. Data represent mean ± SD. **P<0.01.
(TIF)

S5 Fig Effect of miR-202-3p mimic and inhibitor and MMP1 SiRNA on viability of breast cancer cells. MDA-MB-231-TGL cells were transfected with miR-202-3p inhibitor (30 nM) and/or MMP1 SiRNA (30 nM) or negative control. MDA-MB-231-BrM2 cells were transfected with miR-202 mimic (5 nM) or scrambled control and cell viability was assessed my MTT assay. Experiments were carried out three times.
(TIF)

S1 Raw images (PDF)
