# Supplementary material for: Silencing miR-202-3p increases MMP-1 and promotes a brain invasive phenotype in metastatic breast cancer cells
Source: PLoS One. 2020 Oct 1;15(10):e0239292. doi: 10.1371/journal.pone.0239292 (PMC7529272; doi:10.1371/journal.pone.0239292)
Supplement: S1 Table — The clinical micro-RNA array cohort data (GSE37407) which contains global microRNA profiling performed on primary breast tumor samples from 10 patients and their corresponding paired brain metastatic tumors was analyzed with the GEOR tool [33, 36]. Analysis with the GEO2R tool of the top 250 microRNAs differentially expressed between the primary and brain metastatic breast tumors revealed that five microRNAs are downregulated in brain metastatic tumors compared to breast primary tumors and also predicted to target MMP1 using four prediction databases: mirTarBase 7.0, miRanda-mirSVR (microRNA.org), and miRDB, TargetScan 7.2 [42]: miR-202-3p, miR-326, miR-623, let-7c, and miR-145. Three of these micro-RNAs (miR-202-3p, miR-623 and miR-145) are validated to target MMP1 [26, 32, 45]. A significant downregulation of miR-202-3p was also reported by a study by Xing et al., where a micro-RNA profiling was performed on 7 primary tumors and their paired metastatic lesions in the brain [37]. (DOCX) [file pone.0239292.s001.docx]

| **Mature micro-RNAs downregulated in GSE37407 and validated or predicted to target MMP1** | | | | | |
| --- | --- | --- | --- | --- | --- |
| **Validated** | **Predicted** | | | | **Micro-RNAs commonly downregulated in GSE37407 and Xing et al. (36,37)** |
|  | **TargetScan 7.2** | **miRanda-mirSVR (microRNA.org)** | **miRDB** | **mirTarBase 7.0** |  |
| hsa-miR-202 | hsa-miR-202 | hsa-miR-202 | hsa-miR-202-3p | hsa-miR-202 | hsa-miR-202-3p |
| hsa-miR-145 | hsa-miR-326 | hsa-miR-326 | hsa-miR-326 | hsa-miR-145 |  |
| hsa-miR-623 | hsa-let-7c | hsa-let-7c |  |  |  |
|  | hsa-miR-623 |  |  |  |  |

**S1 Table. Mature micro-RNAs downregulated in GSE37407 and validated or predicted to target MMP1.** The clinical micro-RNA array cohort data (GSE37407) which contains global microRNA profiling performed on primary breast tumor samples from 10 patients and their corresponding paired brain metastatic tumors was analyzed with the GEOR tool (33,36). Analysis with the GEO2R tool of the top 250 microRNAs differentially expressed between the primary and brain metastatic breast tumors revealed that five microRNAs are downregulated in brain metastatic tumors compared to breast primary tumors and also predicted to target MMP1 using four prediction databases: mirTarBase 7.0, miRanda-mirSVR (microRNA.org), and miRDB, TargetScan 7.2 (42): miR-202-3p, miR-326, miR-623, let-7c, and miR-145. Three of these micro-RNAs (miR-202-3p, miR-623 and miR-145) are validated to target MMP1 (26,32,45). A significant downregulation of miR-202-3p was also reported by a study by Xing et al., where a micro-RNA profiling was performed on 7 primary tumors and their paired metastatic lesions in the brain (37).
