# Supplementary figures and images for: Silencing miR-202-3p increases MMP-1 and promotes a brain invasive phenotype in metastatic breast cancer cells
Source: PLoS One. 2020 Oct 1;15(10):e0239292. doi: 10.1371/journal.pone.0239292 (PMC7529272; doi:10.1371/journal.pone.0239292)

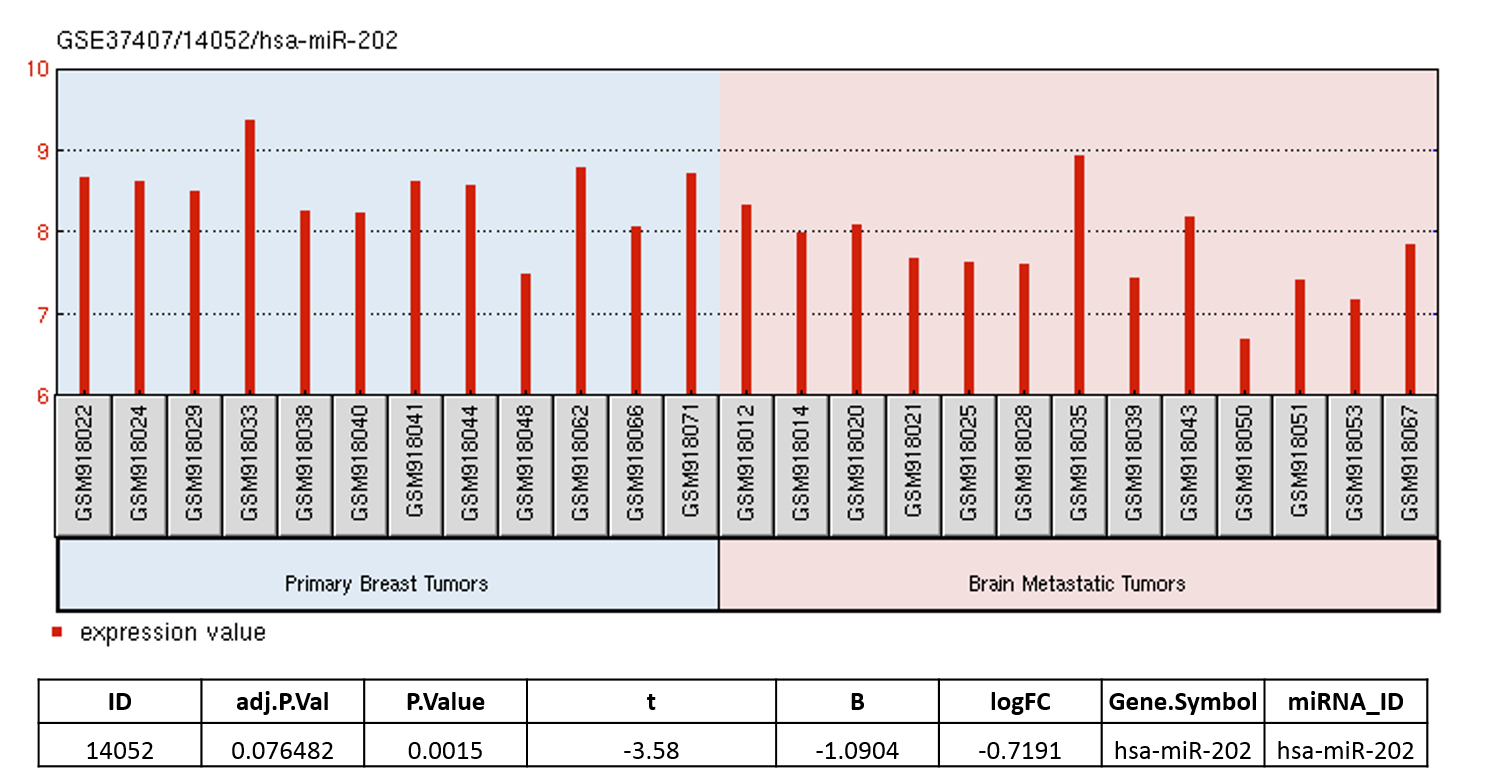

Supplement: S1 Fig — (PNG) [file pone.0239292.s002.png]

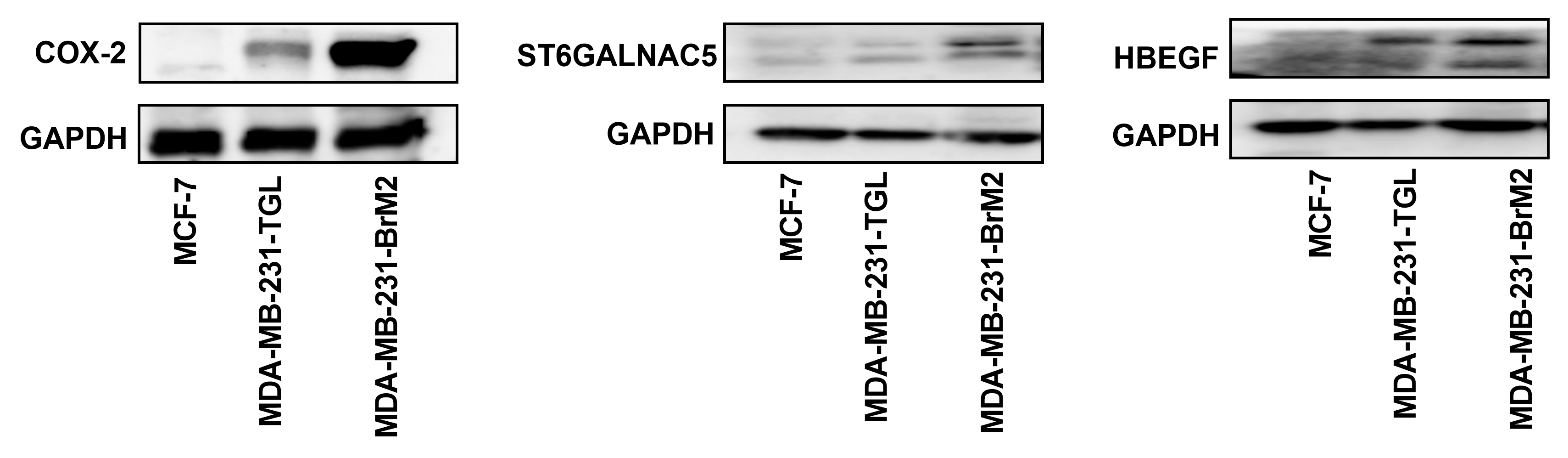

Supplement: S2 Fig — COX-2, ST6GALNAC5 and HBEGF protein expression was assessed by western-blot in three breast cancer cell lines with different metastatic propensities (MCF-7; MDA-MB-231-TGL and MDA-MB-231-BrM2). (TIF) [file pone.0239292.s003.tif]

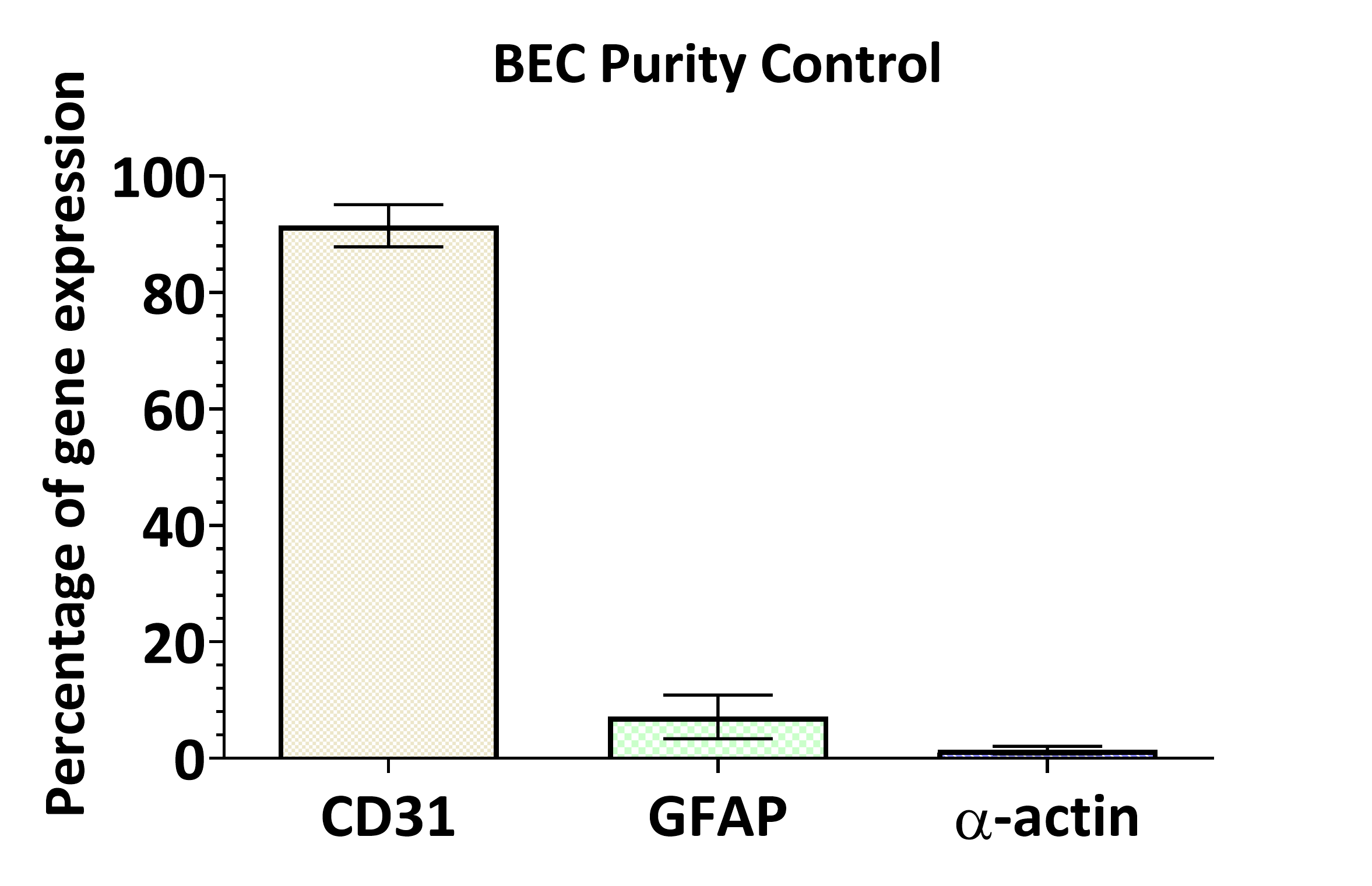

Supplement: S3 Fig — Gene expression of CD31 (marker of endothelial cells), GFAP (marker of astrocytes) and α-actin (marker of pericytes) was measured by real time PCR in primary BECs isolated from mice. (TIF) [file pone.0239292.s004.tif]

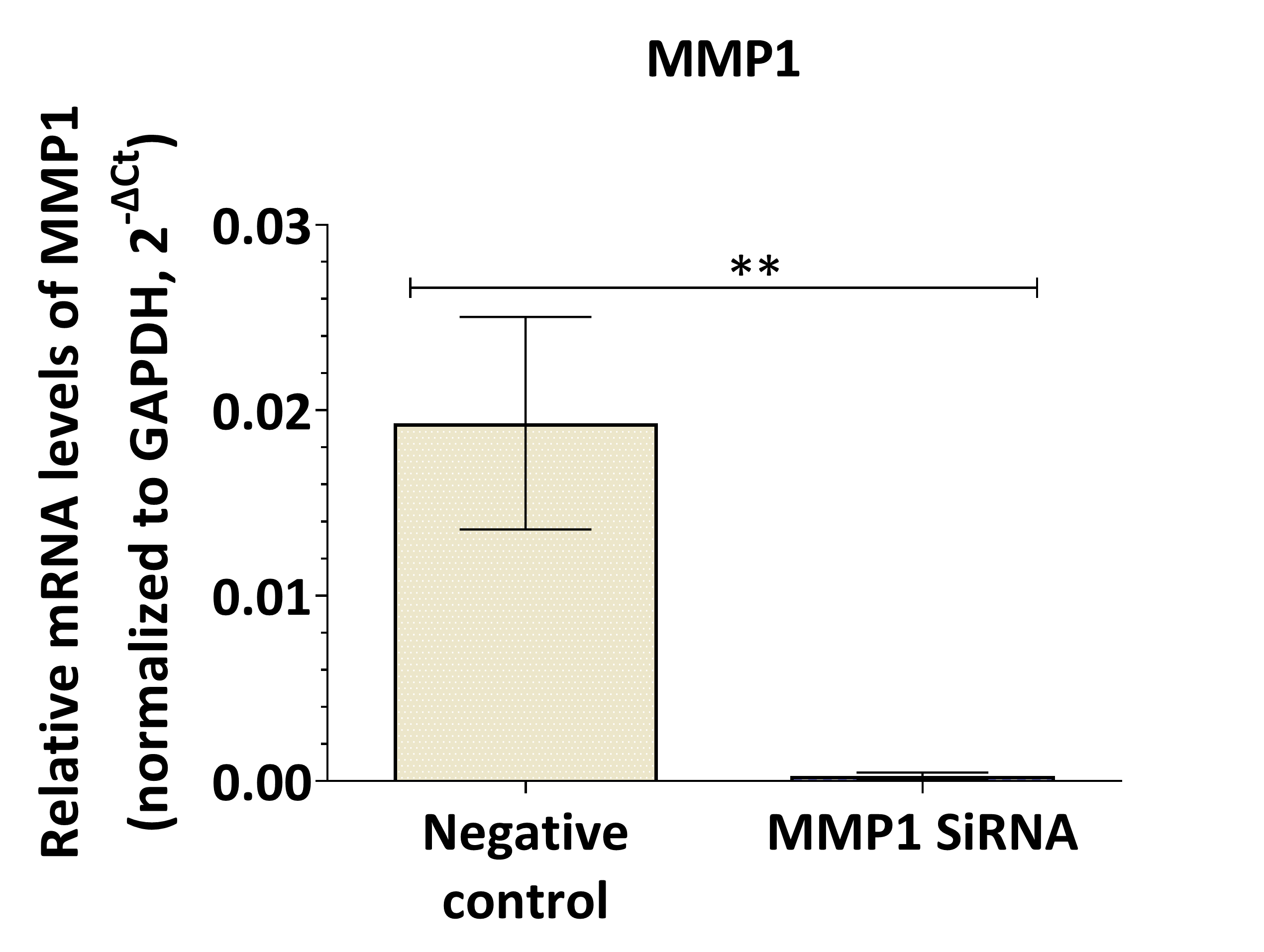

Supplement: S4 Fig — MDA-MB-231-BrM2 cells were treated with MMP1 SiRNA and gene expression was examined by real-time PCR 48 hours later. Experiments were carried out three times. Data represent mean ± SD. **P<0.01. (TIF) [file pone.0239292.s005.tif]

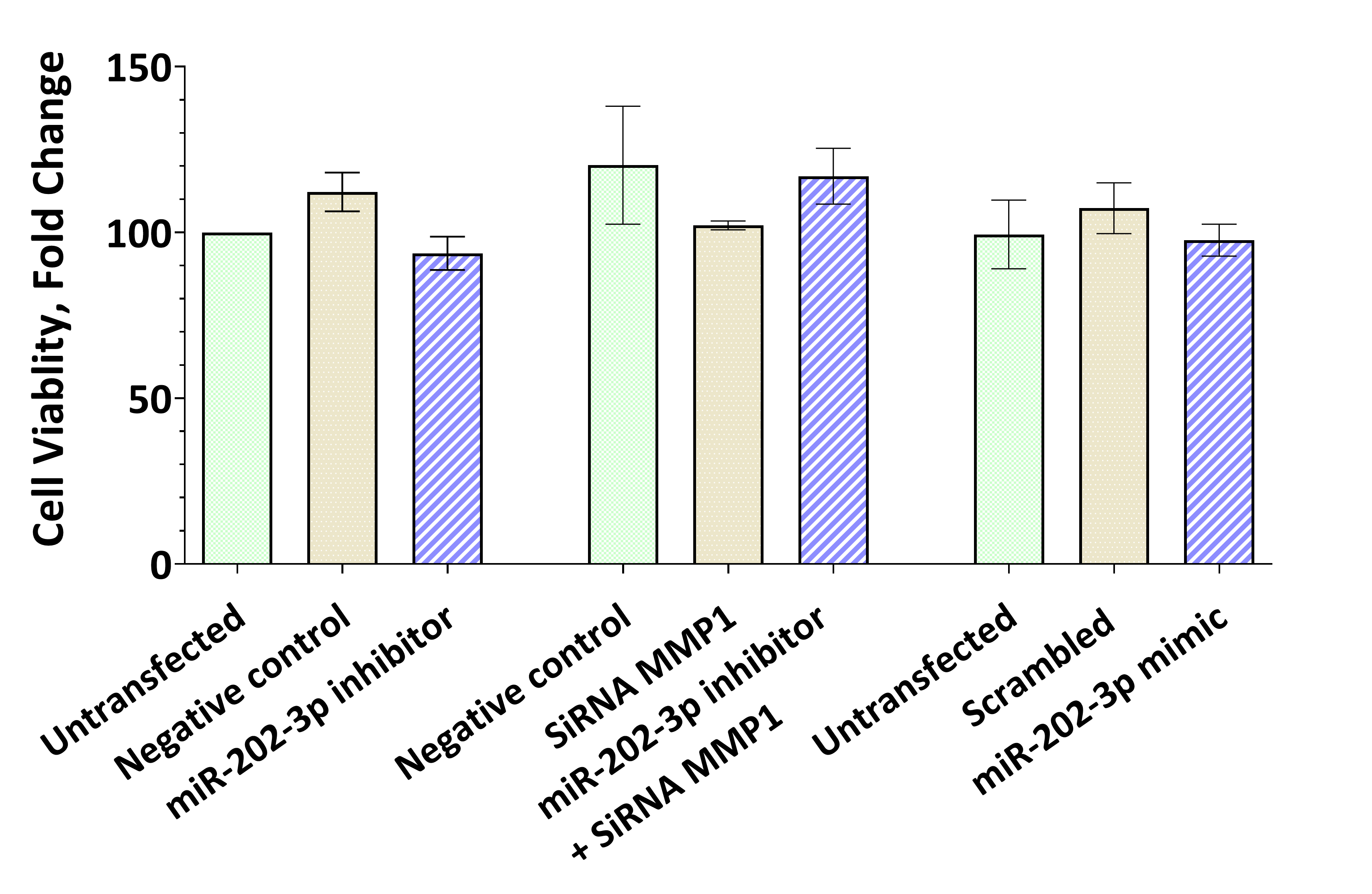

Supplement: S5 Fig — MDA-MB-231-TGL cells were transfected with miR-202-3p inhibitor (30 nM) and/or MMP1 SiRNA (30 nM) or negative control. MDA-MB-231-BrM2 cells were transfected with miR-202 mimic (5 nM) or scrambled control and cell viability was assessed my MTT assay. Experiments were carried out three times. (TIF) [file pone.0239292.s006.tif]
