# Supplementary material for: Silencing miR-202-3p increases MMP-1 and promotes a brain invasive phenotype in metastatic breast cancer cells
Source: PLoS One. 2020 Oct 1;15(10):e0239292. doi: 10.1371/journal.pone.0239292 (PMC7529272; doi:10.1371/journal.pone.0239292)

Silencing miR-202-3p increases MMP-1 and promotes a brain invasive phenotype in metastatic breast cancer cells

Fig 1 F. MMP1expression in breast cancer cell lines

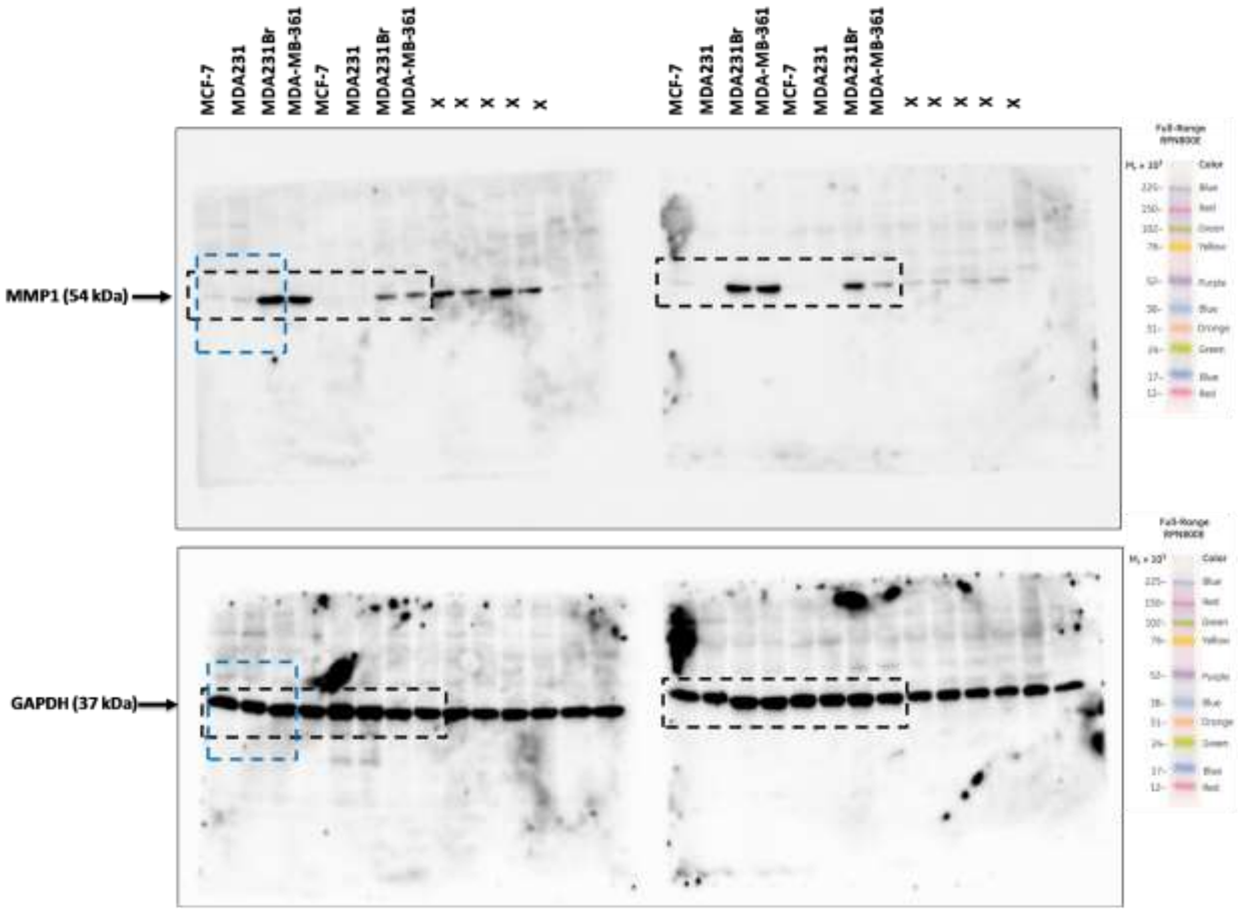

Fig 3C.

MMP1 expression in MDA-MB-231-TGL cells transfected with miR-202-3p inhibitor

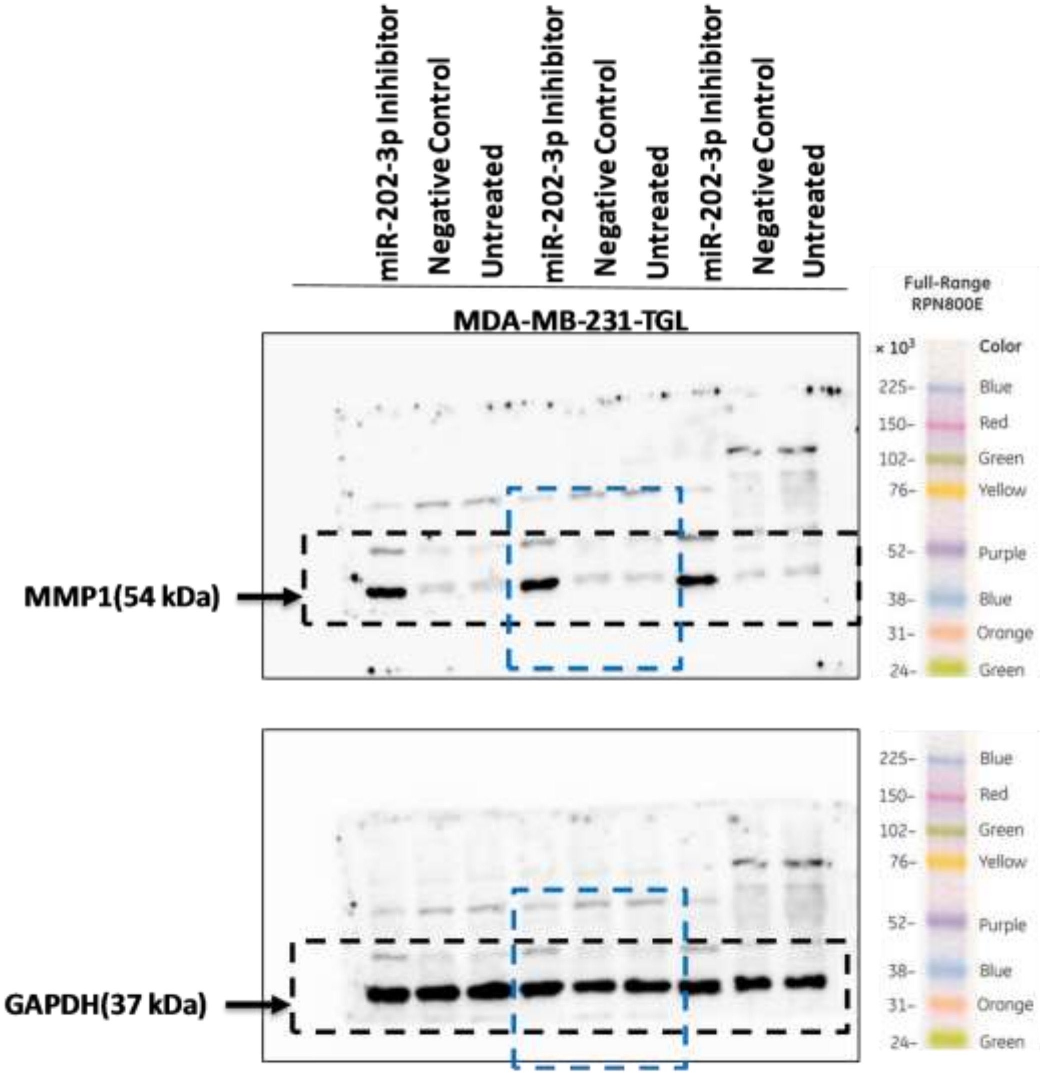

Fig 3F. MMP1 expression in MDA-MB-231-TGL cells transfected with miR-202-3p inhibitor and/or MMP1 SiRNA

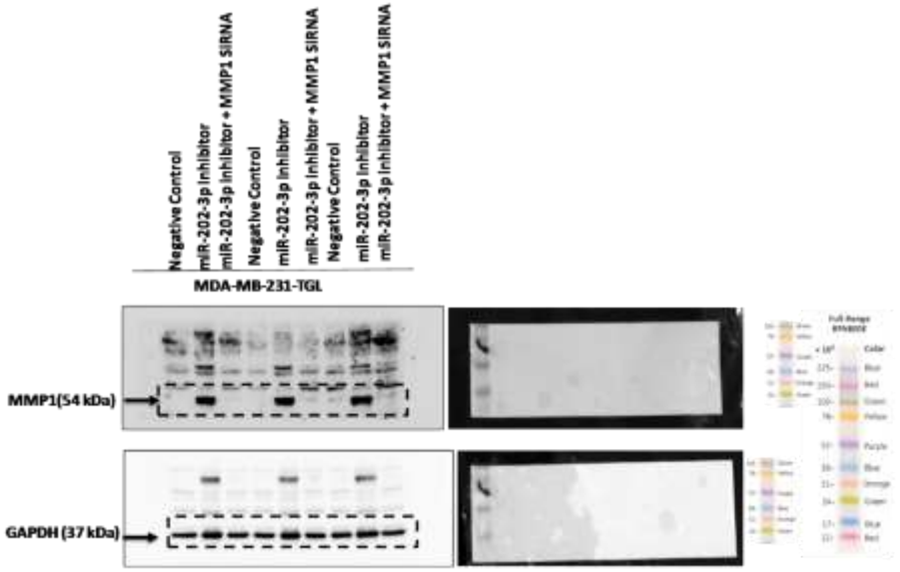

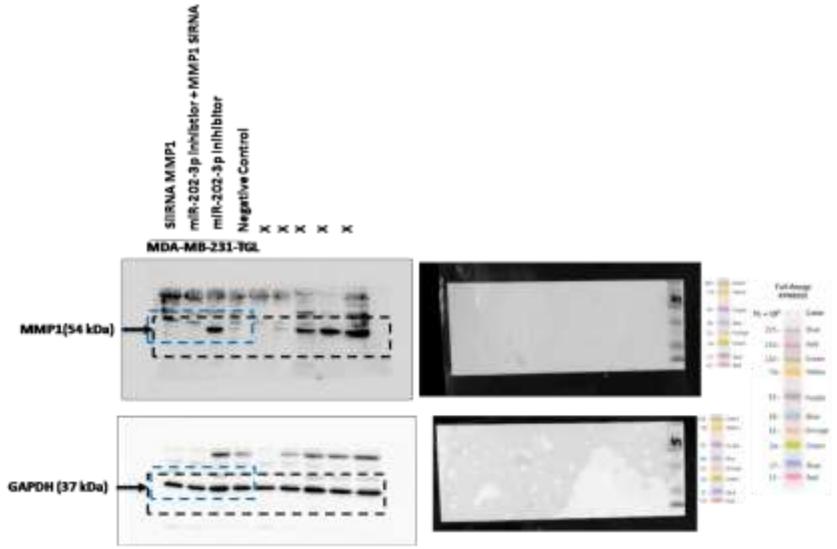

Fig 4D.

Expression of inter-endothelial junctions co-cultured with MDA-MB-231-TGL cells pre-transfected with miR-202-3p inhibitor

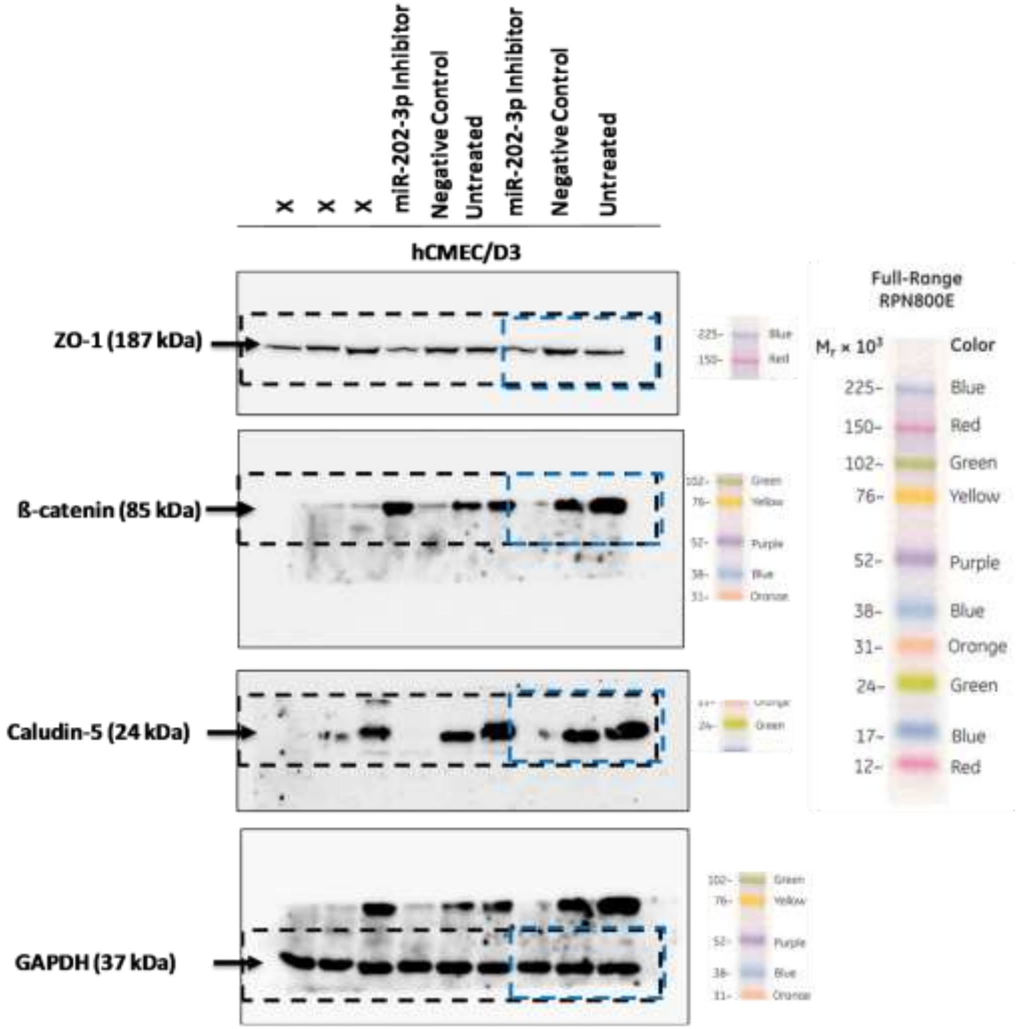

Fig 4G.

Expression of inter-endothelial junctions co-cultured with MDA-MB-231-TGL cells pre-transfected with miR-202-3p inhibitor and/or MMP1 SiRNA

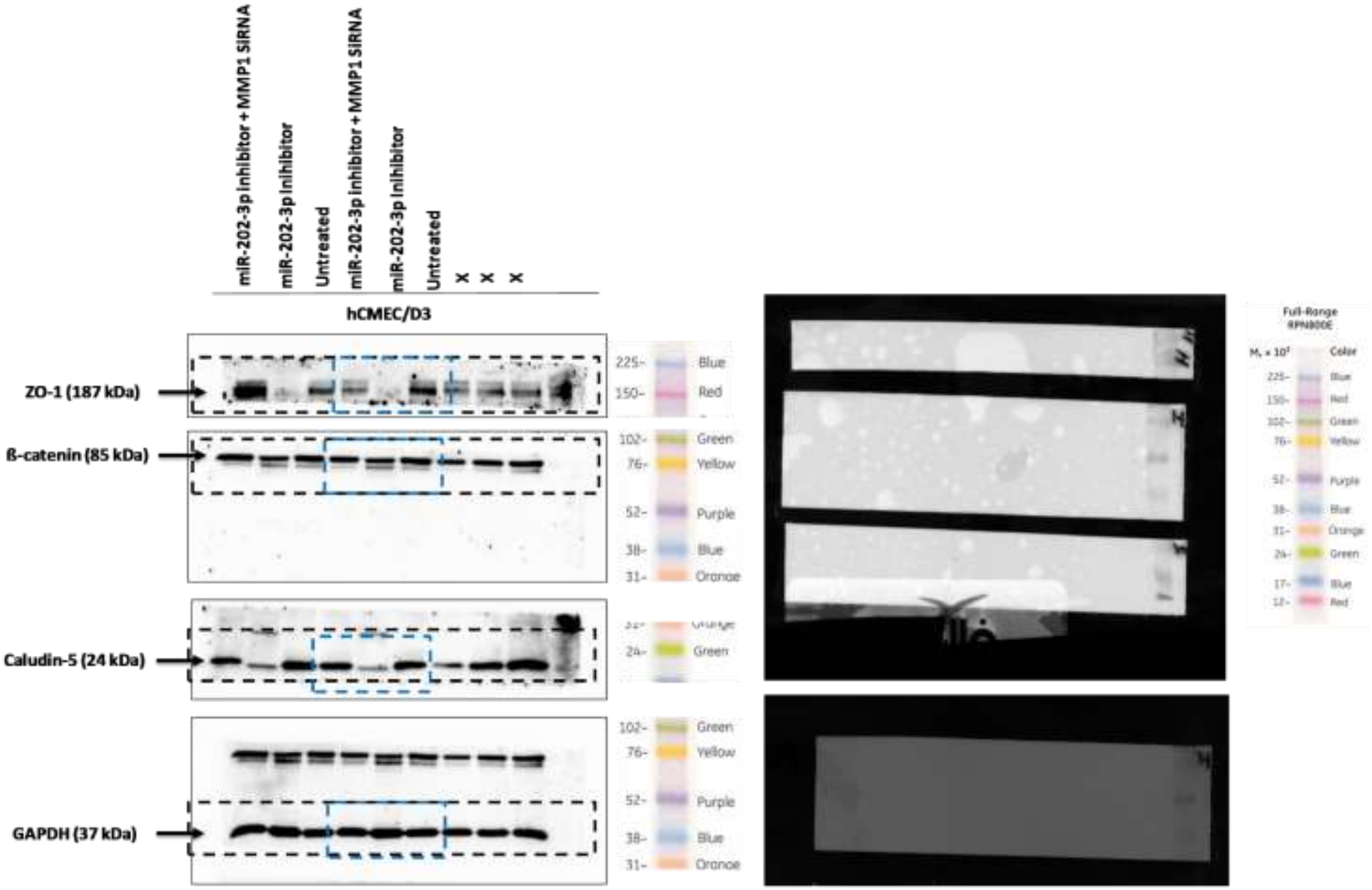

Fig 4G.

Expression of inter-endothelial junctions co-cultured with MDA-MB-231-TGL cells pre-transfected with miR-202-3p inhibitor and/or MMP1 SiRNA

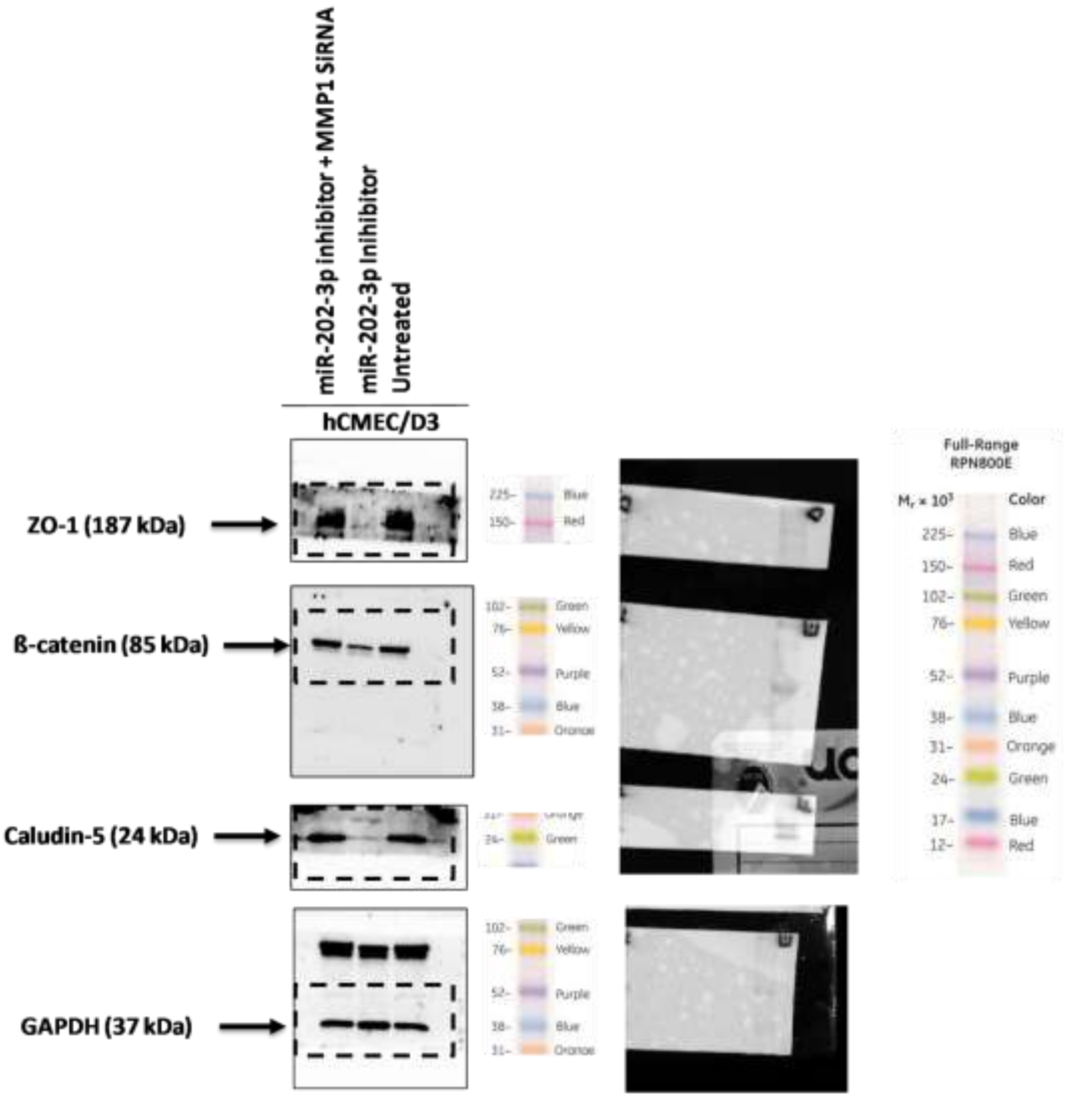

Fig 5C.

MMP1 expression in MDA-MB-231-BrM2 cells transfected with miR-202-3p mimic

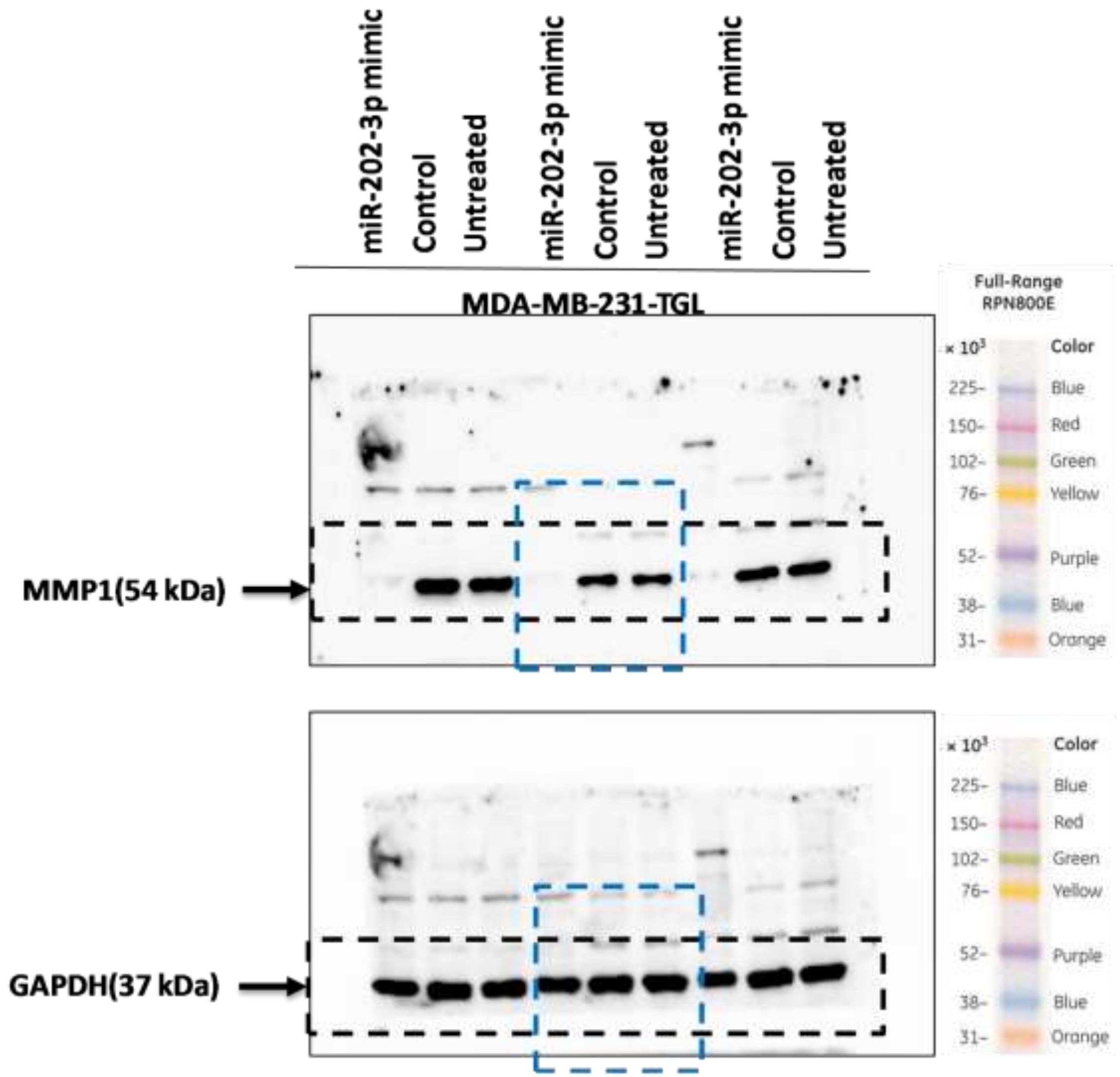

Fig 6E.

Expression of inter-endothelial junctions co-cultured with MDA-MB-231-BrM2 cells pre-transfected with miR-202-3p mimic

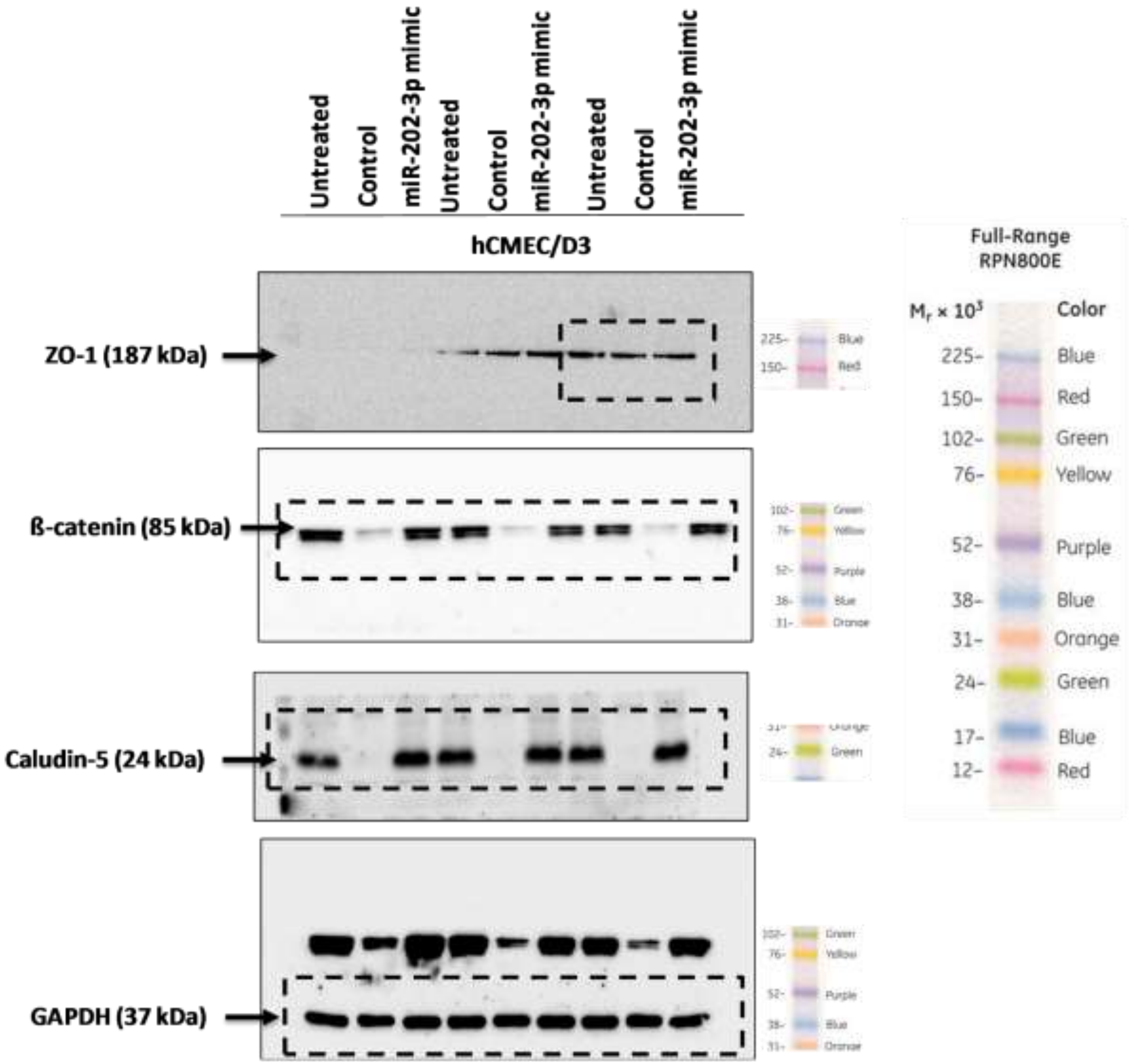

Fig 4D.

Expression of inter-endothelial junctions co-cultured with MDA-MB-231-TGL cells pre-transfected with miR-202-3p inhibitor

Fig 6E.

Expression of inter-endothelial junctions co-cultured with MDA-MB-231-BrM2 cells pre-transfected with miR-202-3p mimic

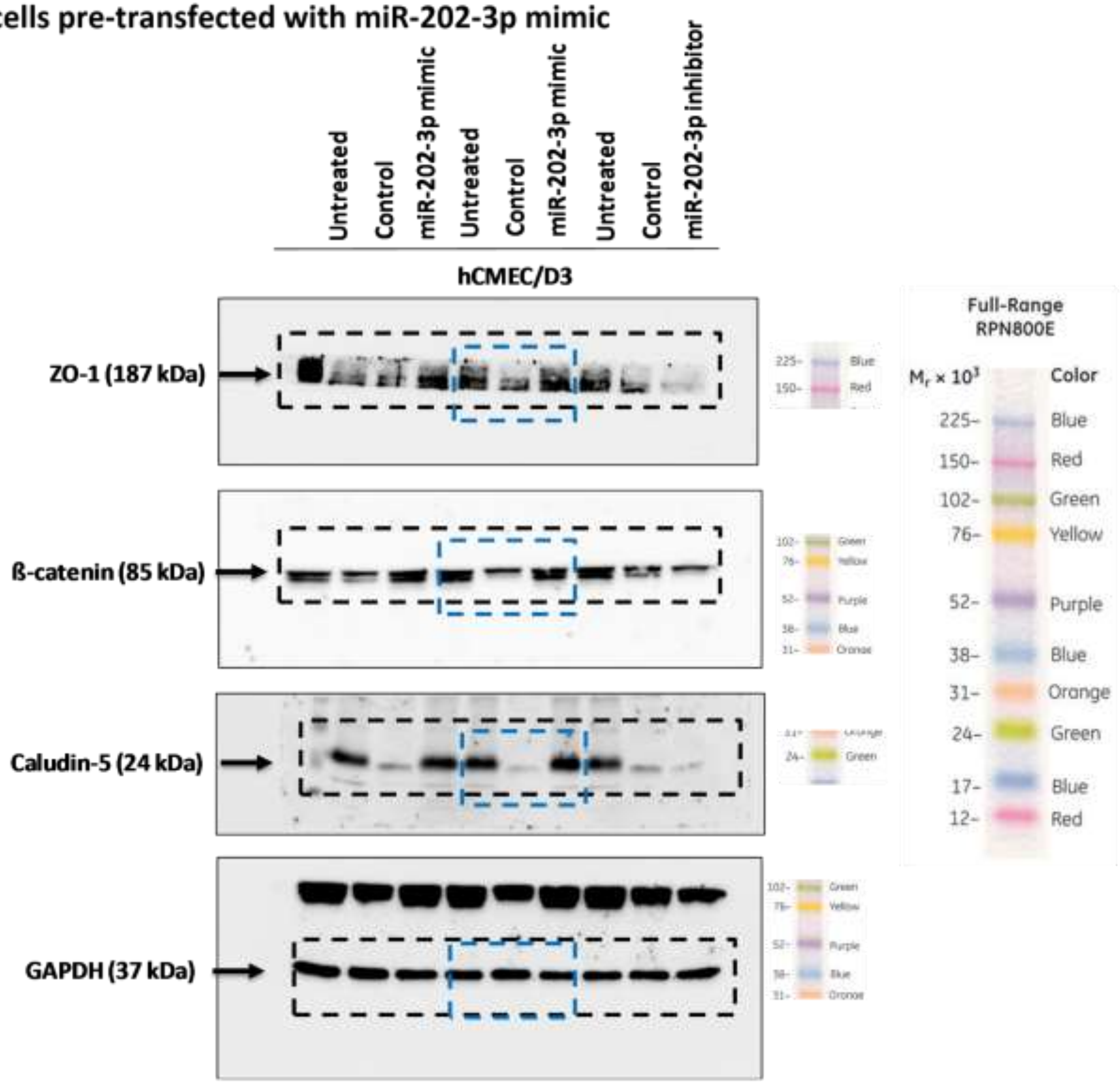

Supplement: S1 Raw images — (PDF) [file pone.0239292.s007.pdf]
